# A Machine Learning Approach to Understand Thermal Desorption Profiles of Levoglucosan from FIGAERO–CIMS

**DOI:** 10.1021/acs.est.5c18488

**Published:** 2026-05-20

**Authors:** Yvette Gramlich, Roman Spahr, Abhishek Upadhyay, Karolina Siegel, Sophie L. Haslett, Radovan Krejci, Karl Espen Yttri, Claudia Mohr

**Affiliations:** † PSI Center for Energy and Environmental Science, 5232 Villigen PSI, Switzerland; ‡ Department of Environmental Systems Science, 27219 ETH Zurich, 8092 Zurich, Switzerland; § Department of Environmental Science, 7675 Stockholm University, Stockholm 11418, Sweden; ∥ 87482 NILU, Kjeller 2027, Norway

**Keywords:** machine learning, chemical ionization mass spectrometry, aerosol thermal desorption, volatility, biomass burning

## Abstract

The Filter Inlet for Gases and AEROsols coupled to a Chemical Ionization Mass Spectrometer (FIGAERO–CIMS) can be used to derive volatility of atmospheric aerosol by using the temperature at thermogram maximum signal (*T*_max_). For complex ambient particle matrices, *T*_max_ of an individual compound often varies, for reasons not fully elucidated. Here, we apply machine learning to study the relation between *T*_max_ of levoglucosan (C_6_H_10_O_5_), a common tracer to identify the influence of biomass burning (BB) in ambient air, and a set of atmospheric and instrumental parameters for an ambient year-long FIGAERO–CIMS data set measured in the Arctic. Using three different modeling approaches, namely, multiple linear regression (MLR), random forest (RF) regressor, and XGBoost regressor, we find that the mass loading on the FIGAERO filter has the highest relevance for variation in *T*_max_ of levoglucosan. On the basis of these results, we suggest controlling the mass collected on the filter for continuous online measurement with the FIGAERO–CIMS if quantitative volatility information is to be gained. More generally, we demonstrate the usefulness of machine learning approaches for characterization of instrumental backgrounds in complex ambient or laboratory data.

## Graphical abstract

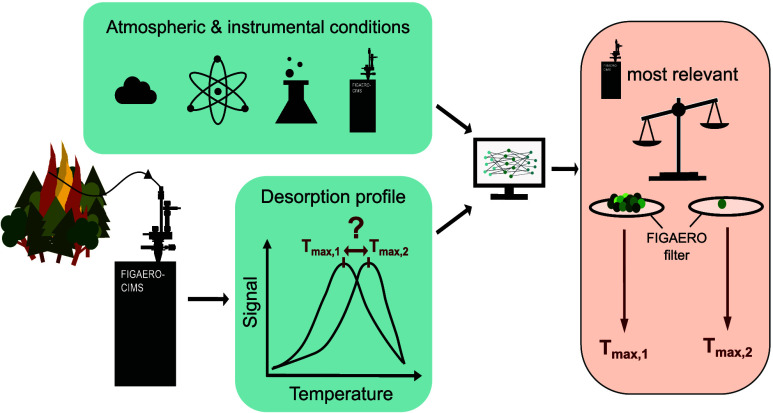

## 1 Introduction

Emissions from biomass burning (BB) are an important source of aerosol particles and gases in the atmosphere.^1^ In the northern hemisphere, regions with enhanced BB activity are Canada, Eastern Europe, and Siberia.^2,3^ While undergoing atmospheric transport, the emissions from these burning activities can reach regions far away from the emission source.^4,5^ Remote places such as the Arctic thus occasionally experience extreme values of aerosol properties, e.g., aerosol mass concentrations that are more than 1 order of magnitude above the background levels.^6,7^ In turn, this has consequences for the local aerosol–cloud and aerosol–radiation interactions and the accelerated warming of the Arctic region.^5,8,9^

A common tracer used to identify BB emissions is levoglucosan (1,6-anhydro-β-d-glucopyranose, C_6_H_10_O_5_), associated with the combustion or pyrolysis of cellulose and hemicellulose.^10–12^ As a semivolatile compound,^13^ it can partition between the gas and the particle phase. When being dispersed in the atmosphere, the ambient conditions can change and the levoglucosan emissions from the source region can get diluted, which consequently has an impact on its contribution to the particulate phase of the BB plume.^14^ In the literature, the lifetime of levoglucosan in the atmosphere spans from a few hours^15^ to several days^10^ up to few weeks,^16^ and depends largely on the OH concentration.

To determine the distribution between the gas and the particle phase of levoglucosan and other secondary organic aerosol (SOA) components, information on their volatility is needed.^17^ A measurement technique suitable to investigate SOA across different volatility ranges is the Filter Inlet for Gases and AEROsols coupled to a Chemical Ionization Mass Spectrometer (FIGAERO–CIMS^18,19^ ). This instrument thermally desorbs aerosol particles collected on a filter for subsequent analysis of desorbed compounds in the mass spectrometer.^20^ Based on the desorption profile (called thermogram, the signal as a function of desorption temperature), the volatility of a compound can be derived by using the temperature of the maximum signal of this curve, *T*_max_.^21–25^ Volatility distributions of the entire particle populations collected on the FIGAERO–CIMS filter can be derived using positive matrix factorization on the thermograms of all compounds, where each resulting factor can be linked to a certain *T*_max_.^26,27^ Another possibility to group thermograms is to use a clustering algorithm based on their similar volatility behavior in the FIGAERO–CIMS, as presented by Li et al.^28^ Further, volatility estimates (i.e., parametrization of saturation concentrations) can be based on the measured molecular composition of compounds.^29–33^

Despite past efforts to investigate the volatility of SOA with FIGAERO–CIMS, aspects remain that require further exploration, especially for ambient measurement data. Factors reported to have an influence on *T*_max_ other than the volatility of a compound include, e.g., thermal decomposition.^23,34–37^ How much decomposition occurs can also be influenced by the rate at which the temperature is increased on the FIGAERO filter.^37^ Further, the viscosity of the collected particles, their size, and the mass loading on the FIGAERO filter can affect *T*_max_.^38–40^

The association of changes in *T*_max_ and derived volatility distributions with ambient conditions or instrumental parameters of the FIGAERO–CIMS remains poorly explored. This is the focus of the present study. The significance of levoglucosan as a BB tracer and the high sensitivity of the reagent ion iodide used in this study^41^ to levoglucosan make it a suitable and relevant molecule to explore variation in thermograms in more detail at different ambient and instrument conditions.

We use three different models, a multiple linear regression model and two machine learning (ML) models, to identify the relevance of a set of meteorological, aerosol physical and chemical, as well as instrumental parameters for *T*_max_ of levoglucosan thermograms obtained from a FIGAERO–CIMS year-long ambient data set. Recently, ML algorithms are becoming an increasingly important tool in atmospheric science to investigate the fate of aerosols in the atmosphere or improve predictions of atmospheric transport models.^42,43^ ML has been applied to FIGAERO–CIMS data to investigate the relation between precursor gases and the volatility of OA.^43^

Thus, this study applies a new approach to ambient FIGAERO–CIMS levoglucosan thermograms with the aim to contribute toward an improved interpretability of ambient measurements of thermograms and derivation of volatility distributions.

## 2 Materials and Methods

The data included in this study were collected during the Ny-Ålesund Aerosol Cloud Experiment (NASCENT).^44^ This Arctic field campaign took place at the Zeppelin Observatory (78.90° N, 11.88° E, 472 m a.s.l.) in 2019/2020 on Svalbard, known for measuring atmospheric parameters for more than three decades.^45^ Except for some larger gaps in the summer months (see Siegel et al.^46^ and Gramlich et al.^47^), the data used here cover the entire year of 2020.

The relationship between a set of meteorological, aerosol physical, aerosol chemical, and instrumental parameters and thermogram *T*_max_ was established by using a simple linear regression model and two machine learning models. The analysis was done using Python 3.11.11.

### 2.1 FIGAERO–CIMS Thermograms

The instrumental setup and operation of the FIGAERO–CIMS at the sampling site are described elsewhere (e.g., Siegel et al.^46^ and Supporting Information of Gramlich et al.^47^). In brief, the instrument was operated behind a whole-air inlet and automatically cycled between the two FIGAERO modes, i.e., the particle collection mode (mode I) and the thermal desorption mode (mode II).^20^ Every third cycle was a blank measurement, where the air was passed over a second filter upstream of the FIGAERO sampling filter. The duration of mode I was 2.5 h, where ambient aerosol particles were collected on the FIGAERO filter. The sampling flow rate was controlled and measured. During mode II, the filter was positioned such that a flow of 2 LPM nitrogen (N_2_) of ultrahigh purity (nitrogen generator from Peak Scientific, UK, model NG5000) could pass over the filter. Within the first 20 min of mode II, the temperature of the N_2_ flow was linearly increased from room temperature to around 200 °C (ramping). Subsequently, the N_2_ flow was kept at around 200 °C for another 20 min (soaking) to ensure full desorption of the collected particles, before the N_2_ flow and thereby the temperature on the filter decreased back to room temperature in the following 20 min. Iodide (I^–^) was used^41^ as the reagent ion in the CIMS. The signal as a function of temperature during the ramping and soaking phases is what we define here as a thermogram. The thermogram signals were normalized to the reagent ion signal, which is referred to hereafter as normalized ion counts per second [ncps].

The analysis procedure of the FIGAERO–CIMS data is described in Gramlich et al.^48^ Data were analyzed using Tofware version V3.2.0 within Igor Pro 7.^49^ The ion at *m*/*z* 288.95786 was identified as I­(C_6_H_10_O_5_)^−^, likely corresponding to levoglucosan. This compound is known for being detected at high sensitivity in iodide CIMS measurements.^50^ However, we cannot fully rule out that the signal of I­(C_6_H_10_O_5_)^−^ can also have contributions from the isomers of levoglucosan, namely, mannosan and galactosan.

FIGAERO–CIMS levoglucosan mass concentrations (see Supporting Information Section S1) derived using a calibration factor determined post campaign in the laboratory showed a reasonable agreement (*R* ^2^ = 0.5) with levoglucosan measurements from weekly offline filter samples (particles smaller 10 μm (PM_10_)) collected at the measurement site, which were analyzed using ultrahigh-performance liquid chromatography coupled to an Orbitrap (see Supporting Information of Gramlich et al.^47^). Levoglucosan was used to separate the data in BB and non-BB influence (see Gramlich et al.^47^ and Supporting Information Section S2).

### 2.2 Environmental and Instrumental Parameters

In total, 18 parameters (Table S1 in the Supporting Information) were part of the analysis, including meteorological, aerosol physical, aerosol chemical, and instrumental parameters, selected to obtain a comprehensive description of the atmosphere, the aerosol particles, and the measurement environment.

The meteorological parameters include ambient temperature (*T*_ambient_), relative humidity (RH), wind direction (WD), and shortwave downward radiation (SWD). *T*_ambient_, RH, and WD at the Zeppelin Observatory were downloaded as 1 h average data from the EBAS database (https://ebas.nilu.no, last access: December 5, 2024).^51^ SWD was obtained as 1 min data in Ny-Ålesund from PANGAEA^52,53^ (https://pangaea.de, last access: November 9, 2024). In addition, the air mass origin was considered by including the fraction of time that back trajectories spent on average over land surface before reaching the station. This fraction was calculated by Freitas et al.^54^

Aerosol physical parameters include particle number and mass concentration (PNC and PM). PNC was recorded with a Differential Mobility Particle Sizer (DMPS), covering an electrical mobility size range from 10 to 800 nm (PNC_0.01–0.8_) and downloaded as 1 h average data from the EBAS database.^55^ Further, the mean and peak diameter, *D*_mean_ and *D*_peak_, were derived from the DMPS data. Mass of particles with an optical diameter between 180 nm and 2.5 μm (PM_0.18–2.5_) was measured with a Fine Dust Aerosol Spectrometer (FIDAS, Model 200 E).

Aerosol chemical parameters considered are the organic mass concentration (ORG), the oxygen-to-carbon ratio (O:C), and the ratio of ORG to PM_0.18–2.5_ (ORG:PM_0.18–2.5_). ORG, measured with an Aerosol Chemical Speciation Monitor (ACSM), was downloaded at 1 h time resolution from the EBAS database. The O:C ratio (signal-weighted average of all organics) was obtained from the FIGAERO–CIMS (see also Gramlich et al.^47^).

The instrumental parameters related to the FIGAERO and its operation (hereafter referred to as FIGAERO parameters) include the mass loading on the FIGAERO filter (*m*_filter_), the mass loading of levoglucosan on the filter (m_I(C6H10O5)‑_), and their ratio (*m*_I(C6H10O5)‑_:*m*_filter_). Furthermore, the mass concentration ratio between levoglucosan and organics (PM_I(C6H10O5)‑_:ORG) was considered, as well as the temperature of the room (*T*_room_) where the FIGAERO was placed, and the difference between the room and the ambient temperature (Δ*T*_room‑ambient_). *T*_room_ was identified as the FIGAERO filter temperature before the start of the ramping. The temperature difference was included to investigate the influence of the sampling conditions. Differences in temperature could lead to changes in particle phase state and its viscosity and subsequently influence the mass transport during thermal desorption.

As all parameters had a different temporal resolution, data were homogenized to the same time resolution, defined by the 2.5 h sampling duration of the FIGAERO–CIMS. This was done using a weighted average of the overlapping time intervals, e.g., if the FIGAERO–CIMS sampling time covered the time period from 17:40 until 20:10, a parameter’s hourly value was weighted with 20/60 between 17:00 and 18:00, 60/60 between 18:00 and 19:00, and 19:00 and 20:00, as well as 10/60 between 20:00 and 21:00. This is because, between 17:00 and 18:00, the FIGAERO–CIMS sampling duration covers 20 out of 60 min in this hour (20/60), while from 18:00 to 19:00, this coverage is 60 out of 60 min (60/60). Then, the average of these weighted values was calculated. For the analysis, only those time periods were considered where all of the 18 parameters had data available. The pairwise correlations among all 18 parameters can be found in the correlation matrix in the Supporting Information, Figure S1.

### 2.3 Target Value Description

The goal of the study is to understand which parameters are the most influential for *T*_max_, as this temperature can in principle be related to the volatility of the aerosol or individual compounds. As such, *T*_max_ is the target value used in the models to investigate the influence of the different parameters. Some of the thermograms showed very smooth desorption profiles, while others showed more noisy desorption curves (see Supporting Information Figure S2). This implies that *T*_max_ values derived from the very smooth desorption profiles have a lower uncertainty than those from less smooth profiles. How the signal-to-noise ratio of each thermogram changes throughout the year of data is presented in the Supporting Information (Figure S3). To attribute higher uncertainty to *T*_max_ for more noisy thermograms, we weighted the *T*_max_ based on the average deviation in absolute signal between each original thermogram and its smoothed version, which we refer to as “noise.” The smoothed version was obtained by applying a Savitzky-Golay filter,^56^ a method to smooth noisy data.^57,58^ This filter was used in the form of the savgol_filter function from the scipy.signal module (SciPy version 1.15.1). By using this filter, a second-degree polynomial fit across a moving window of 7 data points (at a time resolution of 30 s) was applied to the thermograms. For thermograms with high noise (average deviation between original and smoothed version of 3% or larger), the moving window was increased to 15 data points, resulting in a stronger smoothing. The moving window range was chosen based on visual inspection of the comparison between the original and smoothed thermograms.

### 2.4 Modeling Approach

To investigate the relation between all of the 18 parameters and *T*_max_ of the thermograms, three different modeling approaches were used: a multiple linear regression (MLR) model and two machine learning models, Random Forest (RF) Regressor and XGBoost Regressor. The target variable for all of the models was *T*_max_. To take the higher uncertainty of *T*_max_ values of thermograms with higher noise (see Section 2.3) into account, we attributed a lower weight to such *T*_max_ values in the ML models. The weight used here is defined as follows

1 $$\mathrm{weight}=\frac{1}{1+\mathrm{noise}}$$

The MLR is a parametric statistical model to investigate linear relationships among a set of explanatory variables.^59^ The input for the MLR was normalized using MinMaxScaler from the scikit-learn library^60^ (version 1.7.2). Normalization of the data was necessary here to enable an equal contribution from each parameter, such that the model was not driven by large absolute values. To calculate the regression coefficients for the linear model, we used the ordinary least-squares (OLS) method from the python package statsmodels (version 0.14.4).

In contrast to the MLR, the RF and XGBoost regressors were run with the absolute values of the parameters. This is possible because such tree-based models take their decisions based on thresholds instead of the distance between points.^61,62^ As RF and XGBoost handle nonlinearities, robustness, and correlated features slightly differently, the inclusion of both regressors provides higher confidence about the underlying relationships. RF captures nonlinear relationships using many independent decision trees and achieves robustness by averaging their predictions, while also reducing issues from correlated parameters through random selection of parameters.^61^ In contrast, XGBoost builds trees sequentially, where each tree learns from previous errors and achieves robustness through focus on real patterns instead of noise while prioritizing the most important parameters during training.^62^ The RF Regressor was run using the RandomForestRegressor class from the scikit-learn library^60^ (version 1.7.2). The XGBoost Regressor was run using the XGBRegressor class from the xgboost package (version 3.1.2). Target value *T*_max_ was weighted by the above-mentioned *weight* (eq 1) by using the sample_weight argument. For both regressors, the data were divided into 80% for training and 20% for testing using the train_test_split function from the scikit-learn library^60^ (version 1.7.2). For both models, the best hyperparameters were obtained by using RandomizedSearchCV and GridSearchCV from the scikit-learn library^60^ (version 1.7.2). In a first step, the broad parameter space was determined using Randomized Search, and in a second step, the fine-tuning was done using Grid Search. The hyperparameters of the RF Regressor were optimized for the best performance, which was achieved for a training with a forest of 100 trees, a maximum depth of 20, a minimum sample split of 4 and a minimum of 2 sample leaves. For the XGBoost Regressor, the best performance was obtained with 200 trees, a maximum depth of 5 and a learning rate of 0.025. As a measure of the performance, the coefficient of determination (*R* ^2^) and the root mean squared error (RMSE) were used, both from the scikit-learn library^60^ (version 1.7.2).

For the evaluation of the RF Regressor and the XGBoost Regressor regarding the importance of each parameter for *T*_max_, the permutation feature importance (PFI)^60,61^ and the Shapley additive explanations (SHAP)^63^ values were used. The PFI was obtained from the scikit-learn library^60^ (version 1.7.2), and the SHAP values were obtained from the SHAP library (version 0.50.0). While PFI relates the performance of the model to random permutations in the individual parameters, SHAP indicates the magnitude of the average contributions of the individual parameters. This means that PFI gives insights into the reliability of the model prediction and SHAP provides information on the influence of the range of the parameter values on the model prediction. Hence, the consideration of both PFI and SHAP allowed us to gain a robust interpretation of the regressors.

## 3 Results and Discussion

### 3.1 Thermogram Properties and Variability

In total, 443 levoglucosan thermograms were part of the analysis (Figure 1), covering a range of *T*_max_ from 78.0 to 191.3 °C (average 131.4 °C (±28.1 °C)). Out of these, 37 thermograms were measured during BB events from lower latitudes,^6,8^ which were identified following the definition by Gramlich et al.^47^ As expected, the highest absolute signals (Figure 1a) at *T*_max_ occur for the thermograms measured during the BB events, while the non-BB event thermograms show lower absolute signals at *T*_max_. The average absolute signal at *T*_max_ for the BB event thermograms is 717 ions s^–1^ (±383 ions s^–1^, median: 635 ions s^–1^), while for the non-BB events, this average is 588 ions s^–1^ (±315 ions s^–1^, median: 535 ions s^–1^). Consequently, the data set contains clean and more polluted episodes. This provides a good variety of conditions to investigate the relation of thermogram shape and *T*_max_ to other ambient and instrument-related parameters. The thermograms from the cleaner episodes exhibit still signals above the background (see blank thermograms in Supporting Information Figure S4): While the average maximum signal of all of the non-BB event thermograms is 588 (±315, median: 535) ion s^–1^, the corresponding blanks are much lower with an average of 21 (±18, median: 17) ion s^–1^. This only modest difference between non-BB and BB thermogram signals could be related to the BB event definition, which includes only extreme events. This implies that non-BB conditions can still be influenced by residential wood burning, especially in January and February when long-range transport from the midlatitudes leads to the buildup of Arctic haze.^64^ This contributes to overall higher background conditions that are part of the non-BB thermogram data.

**Figure 1:**
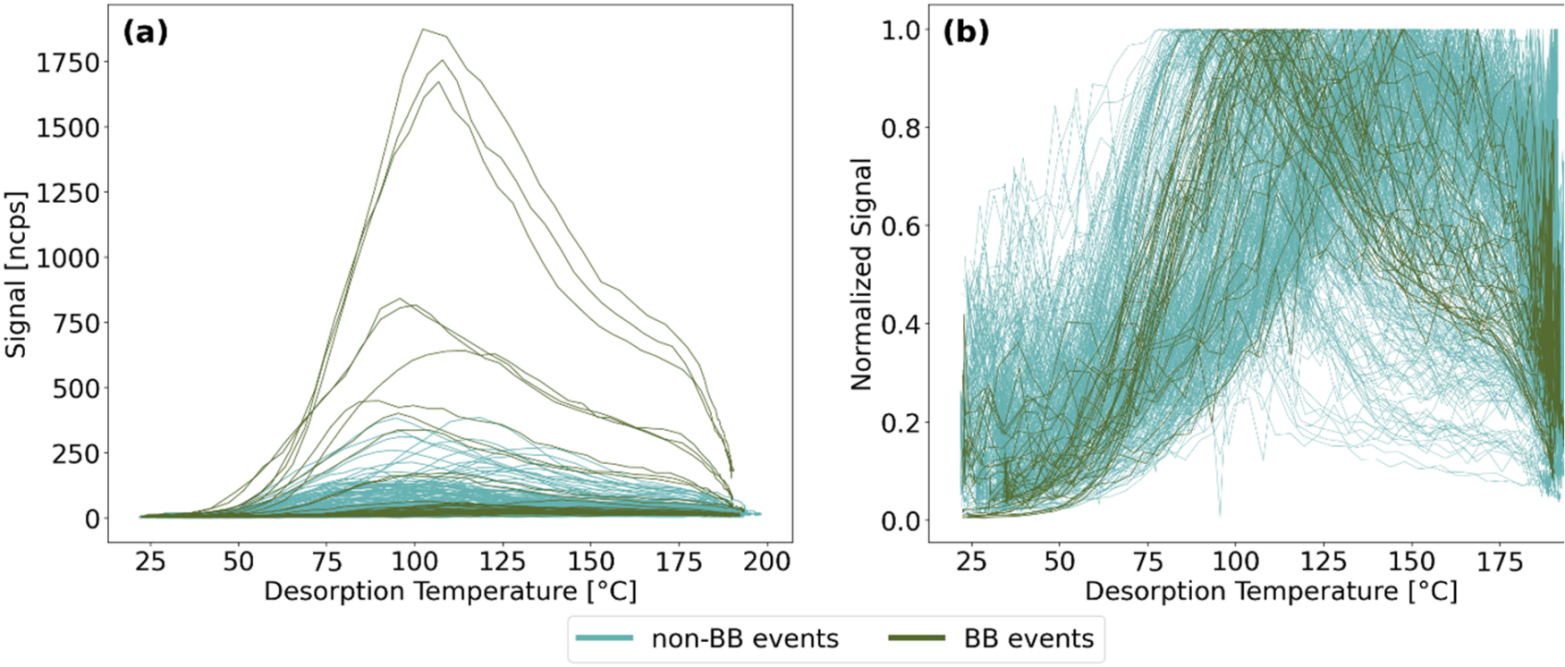
Overview of the levoglucosan thermograms measured in Svalbard during 2020 included in this study as (a) absolute signal and (b) normalized to maximum signal. Those measured during BB events (*n* = 37) are colored in dark green while those from the remaining part of the year (non-BB events, *n* = 406) are presented in blue.

The levoglucosan thermograms normalized to their maximum signal are presented in Figure 1b to visualize the variety of *T*_max_ of the individual thermograms of the entire data set. For the BB event thermograms, the maximum signal occurs on average at 119.4 °C (±19.4 °C, median: 114.3 °C), and for the non-BB events at a slightly higher *T*_max_ with an average of 132.5 °C (±28.6 °C, median: 132.7 °C). Given the relationship between signal and noise, the thermograms that exhibit a larger noise (and lower signal) were observed during non-BB event times.

To understand the variability of the thermogram *T*_max_, we grouped the thermograms in six categories based on their *T*_max_. For this purpose, we split the *T*_max_ values of all thermograms in six bins of equal bin width (Figure 2). This grouping shows underlying differences in the thermogram *T*_max_ across the measurement year. As such, the thermograms in Category 1 (*T*_max_ (78.0, 97.0] °C) have, on average, the lowest *T*_max_ ((89.3 ± 4.8) °C), while those that fall in Category 5 (*T*_max_ (153.6, 172.5] °C) have the highest *T*_max_ ((162.1 ± 4.6) °C). Thermograms from BB events can be observed in several categories, but most of them fall in Category 2 (*T*_max_ (97.0, 115.8] °C), in which the average *T*_max_ is 107.8 °C (±4.9 °C). Thermograms in Category 6 are characterized by high noise, do not exhibit a clear *T*_max_, and resemble rather the blank signal. Hence, they were most likely measured during very clean conditions. As the average *T*_max_ increases from Category 1 toward higher categories, the apparent volatility decreases in the same direction. During the entire measurement period, the same ramping protocol was used and the heating flow during the desorption was constant (values were recorded). With a mass resolving power of the CIMS of ∼5000 *m*/Δ*m*, the I­(C_6_H_10_O_5_)^−^ peak at nominal *m*/*z* 289 could clearly be resolved. This makes instrumental artifacts leading to broad *T*_max_ range rather unlikely. Instead of using a weight factor for *T*_max_ (Section 2.4), filtering the thermograms based on their signal-to-noise ratio or the quality of the fit of the smoothed curve might have been other options to improve the determination of *T*_max_.

**Figure 2:**
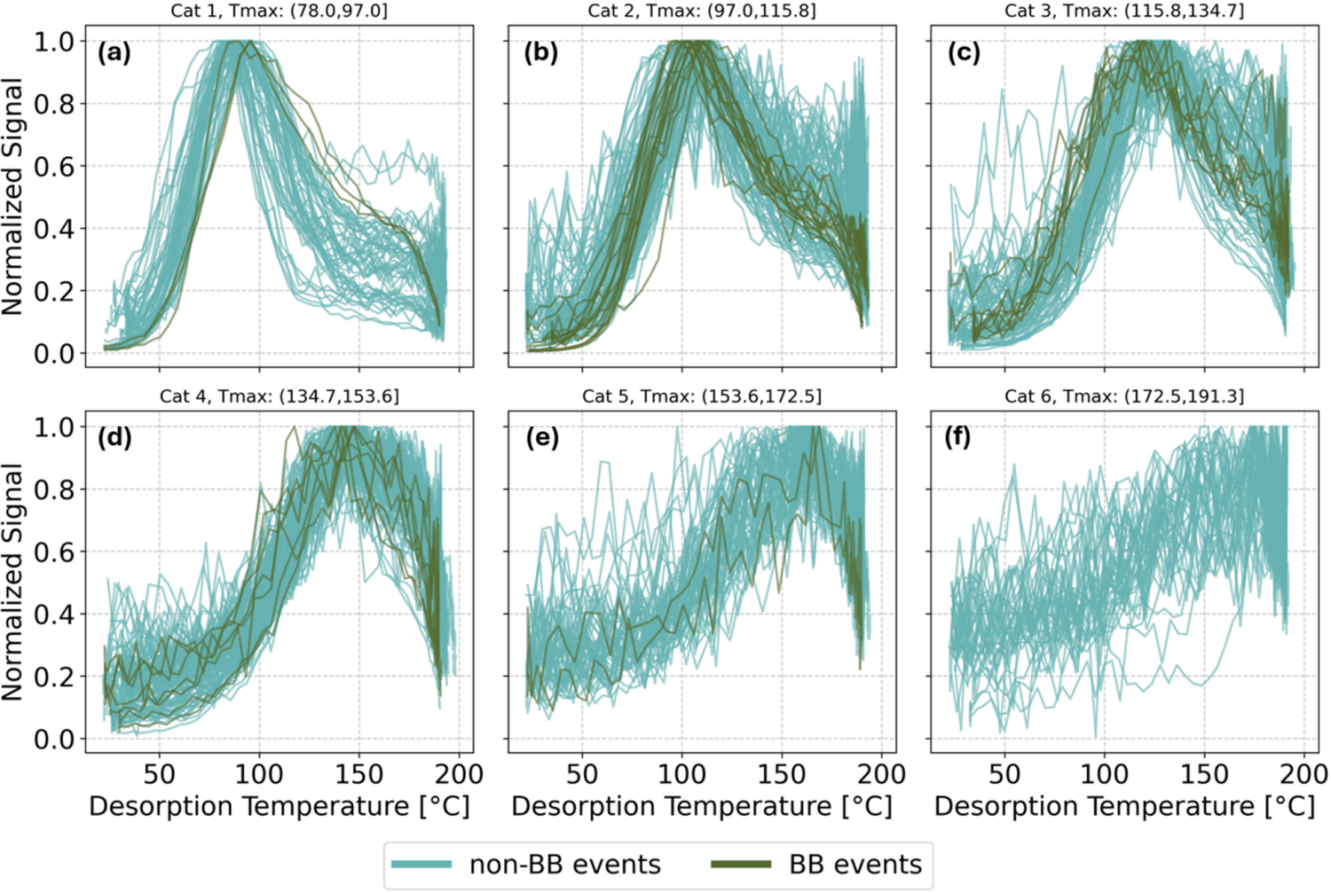
Levoglucosan thermograms used in this study normalized to their maximum signal grouped in six different categories (Cat 1–6, a–f) based on their *T*_max_. The color coding of the thermograms indicates those related to non-BB events (blue) and BB events (dark green). The *T*_max_ interval is indicated in the subpanel titles, in °C.

The differences in *T*_max_ of levoglucosan thermograms across the categories suggests that the apparent volatility behavior of levoglucosan can change in the FIGAERO–CIMS (assuming that the signal of the observed ion I­(C_6_H_10_O_5_)^−^ is uniquely from levoglucosan without interference from a different isomer), complicating the relationship of volatility and *T*_max_ of individual compounds postulated for this technique.^20,23^ To the best of our knowledge, the literature reported no *T*_max_ values for pure levoglucosan from FIGAERO–CIMS. Thermograms of I­(C_6_H_10_O_5_)^−^ from offline filter samples in winter in a German city (Stuttgart) show a *T*_max_ range of approximately 80 to 110 °C, one sample even as low as approximately 55 °C.^19^ In our case, we observe these *T*_max_ values in the thermograms classified as Categories 1 and 2. Graham et al.^32^ compared volatilities of biogenic SOA derived from temperature-dependent particle evaporation in a volatility tandem DMA (VTDMA) to those from FIGAERO–CIMS thermograms. They observed that lower fractions remaining in the VTDMA (i.e., particle with generally higher volatility) coincide with lower *T*_max_ in the FIGAERO–CIMS thermograms, i.e., a qualitative agreement.

A recent laboratory study by Zhang et al.^65^ used a thermodenuder and found that the volatility behavior of levoglucosan depends on its mixing state: as a pure substance, it is less volatile than when mixed with polyethylene glycol or BB primary OA. Although we cannot directly probe the mixing state of individual particles given the FIGAERO–CIMS samples bulk particle mass, we can make the simple assumption for our data that the smaller the ratio of *m*_I(C6H10O5)‑_:*m*_filter_, the more mixed our particles are. The time series of these two ratios (*m*_I(C6H10O5)‑_:ORG and *m*_I(C6H10O5)‑_:*m*_filter_) show elevated values in the winter months compared to those in the summer months (Supporting Information Figure S5). This probably reflects the enhanced transport from lower latitudes during the wintertime, where also BB emissions are transported to the Arctic region.^66^ Obviously, given that our data are ambient samples and the influence of local sources is limited, it is safe to assume that the particles analyzed here are internally mixed and levoglucosan never present as a pure substance; hence, the mixing state may play a minor role in *T*_max_ variation in our data. We discuss this further in Section 3.2.

Very high *T*_max_ values have also been connected to thermal decomposition during the heating process of the particles on the FIGAERO–CIMS filter. A range of previous studies have discussed this effect in the FIGAERO–CIMS, visible in the thermograms either as a second mode or at a much higher than expected *T*_max_ for a certain compound.^34–36,38,67^ As thermograms with high *T*_max_ (Category 5) show a clearly defined peak with declining signal after the peak and do not exhibit multiple peaks nor strong tailing, thermal decomposition can most likely be excluded. This suggests an actual desorption driven behavior and other factors such as matrix effects, particle phase state, or the presence of isomers could be more likely factors for high *T*_max_ values. As noted earlier, isomers of levoglucosan cannot be distinguished using FIGAERO–CIMS. In our previous study,^47^ the comparison of FIGAERO–CIMS levoglucosan mass loadings with those from offline filter samples analyzed with Orbitrap showed a weaker correlation during non-BB episodes (*r* ^2^ = 0.3, as opposed to *r* ^2^ = 0.9 for BB episodes, Figure S2b) in Gramlich et al.^47^ Together with the observation that BB thermograms are predominantly grouped within three of six categories in our clustering analysis, this suggests that isomers might potentially contribute to the signal at I­(C_6_H_10_O_5_)^−^ and thus the observed wide range of *T*_max_. Regular calibrations of levoglucosan and its isomers could have helped to reduce this uncertainty but were unfortunately not possible due to the mostly remote operation of the instrument.

Overall, the thermograms from one year of ambient measurements of levoglucosan showed a range of *T*_max_ values. In the following sections, we apply different modeling approaches including machine learning to understand what factors influence *T*_max_ and thus complicate the *T*_max_–volatility relationship of an individual compound (in this case, levoglucosan).

### 3.2 Influence of Individual Parameters on the Thermogram Shape

As the aim of this study is to understand the relation between *T*_max_ variation of the levoglucosan thermograms and a set of different instrumental and ambient parameters, we compare the predicted importance of 18 parameters for *T*_max_ obtained from the three models in a normalized manner. For the two ML models, the predicted importance refers to the model runs of the 20% of the data that were not part of the training data. For the MLR, all data were considered. Further, for the ML models, *T*_max_ values were weighted according to eq 1 (Section 2.4). The results from the individual models can be found in the Supporting Information (Section S8). A comparison of results without downweighing *T*_max_ is also presented in the Supporting Information (Section S9).

Model performance was evaluated by their *R* ^2^ and RMSE. Here, *R* ^2^ indicates how well the model can capture the underlying patterns of the parameters in relation to *T*_max_. In a range from −1 to 1, a model captures all patterns when *R* ^2^ = 1, while negative values indicate a failure to capture underlying patterns. The RMSE indicates how far, on average, the model predictions of *T*_max_ are from the actual *T*_max_. Due to the normalization of the parameter values for the MLR, the reported unit for the RMSE of the MLR is dimensionless, while for RF and XGBoost the unit of RMSE is °C. The MLR performs with *R* ^2^ = 0.4, RMSE = 0.19. This is overall a good performance for ambient data,^68^ but it also suggests nonlinear relations in the data set. To capture such nonlinear relations, the two machine learning approaches were used. Their performance is better when compared to the MLR. The RF Regressor performs with *R* ^2^ = 0.65, RMSE = 17.24 and the XGBoot Regressor similarly with *R* ^2^ = 0.66, RMSE = 17.22.

Figure 3 shows the normalized importance of all 18 parameters for *T*_max_ variation as given by the three models. The parameters are separated into four groups: meteorological, physical, chemical, and FIGAERO parameters. The two evaluation parameters used for the ML models are PFI and SHAP, which are presented individually in Figure 3a,b, respectively. Both evaluation parameters indicate which parameter is most important for the prediction of *T*_max_ but from different perspectives. A high importance of a parameter according to PFI means that the model relies on this parameter to predict the *T*_max_ correctly. If the values of this parameter were randomly changed while keeping the other parameter values in place, then the model would not predict *T*_max_ correctly anymore. SHAP can be expressed as global importance and as importance per prediction. The importance according to SHAP in Figure 3b shows how much a parameter impacts the prediction of *T*_max_ on average (global importance). The directions in which high and low values of a parameter influence each individual prediction of *T*_max_ are shown in Figure 4. The normalized importance values from the MLR refer to the regression coefficients. The normalized importance in Figure 3 should be interpreted as follows: The higher the normalized importance of a parameter is, the higher its influence on *T*_max_. Similarly, if the normalized importance of a parameter shows a value close to zero, then its influence on *T*_max_ is negligible.

**Figure 3:**
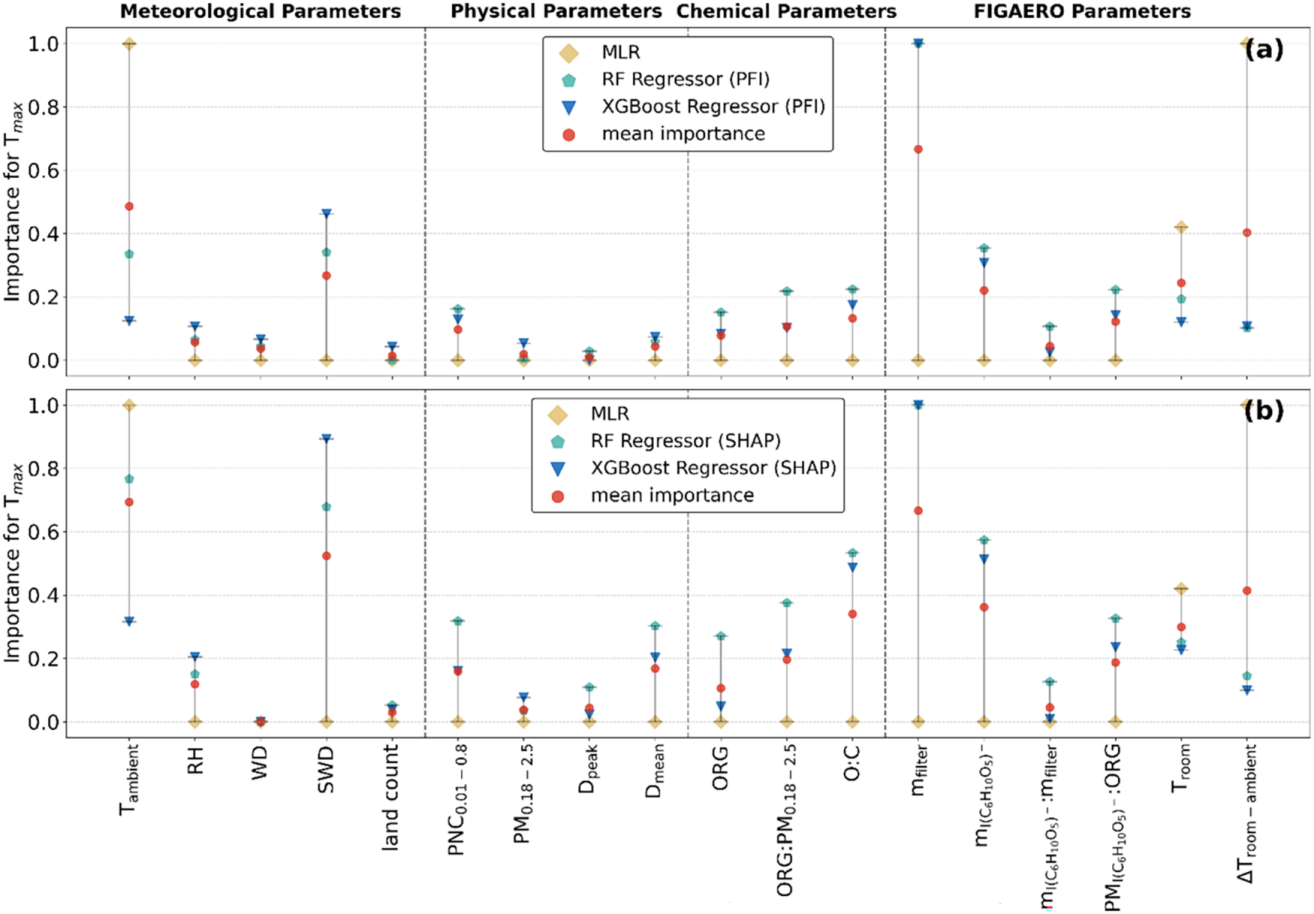
Comparison of the normalized importance of parameters for *T*_max_ variation, grouped into the categories Meteorological Parameters, Physical Parameters, Chemical Parameters, FIGAERO Parameters (for details see Supporting Information Table S1), retrieved from the three different models. To make the models comparable, the importances of the parameters per model were normalized using the MinMaxScaler from the scikit-learn library.^60^ (a) shows the normalized importance of the permutation feature importance (PFI), while (b) shows the normalized importance of the SHAP values. In (a) and (b), the MLR values refer to the normalized MLR regression coefficients. The mean importance indicates the average over all of the models, for each parameter, respectively.

**Figure 4:**
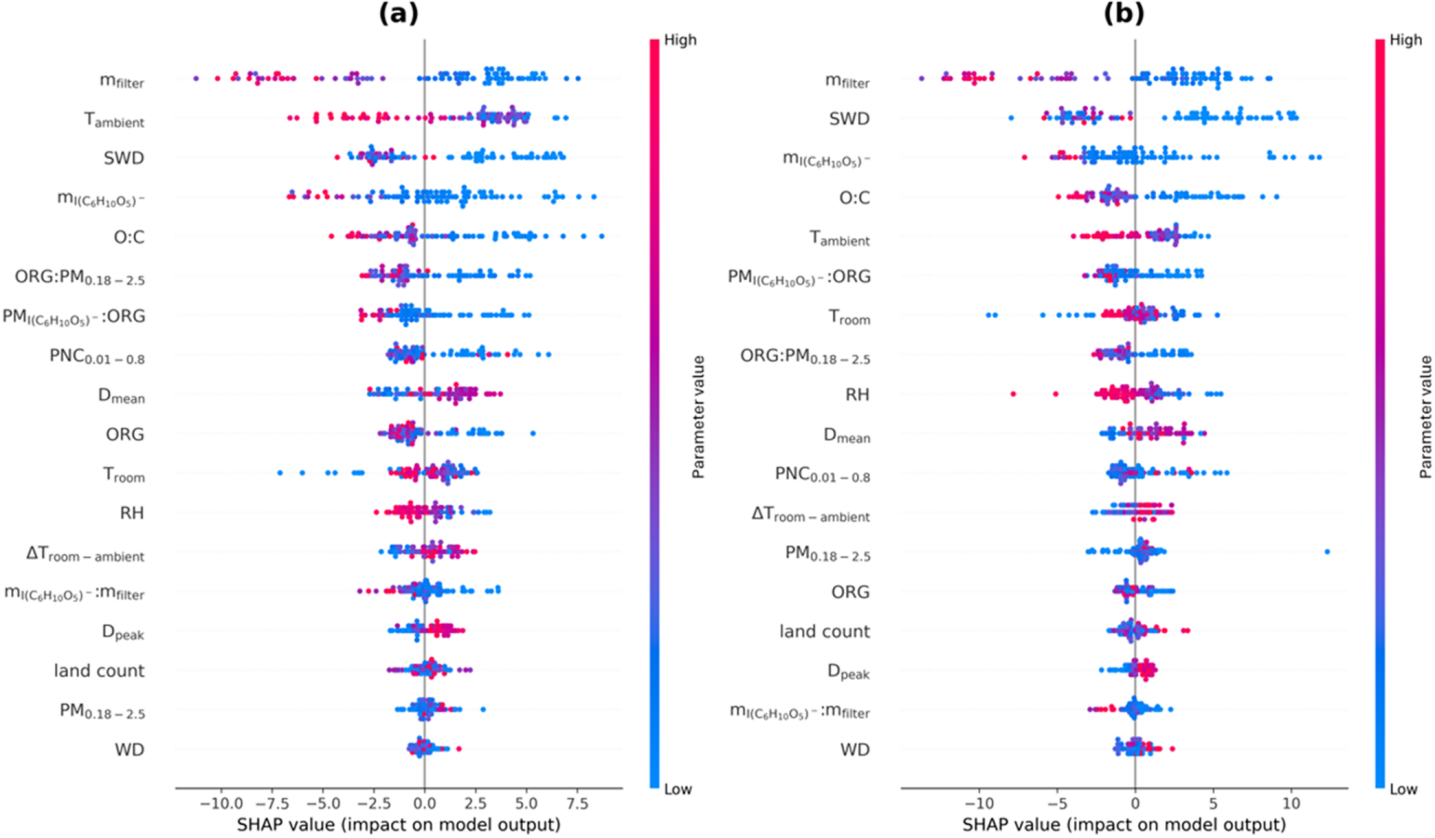
SHAP summary plot for (a) RF Regressor and (b) XGBoost Regressor. From top to bottom, the parameters are sorted according to their overall importance according to SHAP. The dots represent each one prediction of *T*_max_, corresponding to one FIGAERO–CIMS filter sample, and the colors indicate the values of the parameters (high parameter values: red, low parameter values: blue). Negative values on the *x*-axis indicate a decrease in *T*_max_, while positive values on the *x*-axis indicate an increase in *T*_max_.

The relative importance of the different parameters for *T*_max_ variation are largely similar between PFI and SHAP for RF and XGBoost Regressors (Figure 3). This provides high confidence for the ML model predictions; hence, for the underling relation between *T*_max_ and the different parameters. For both evaluation parameters, both models show the largest importance for instrumental parameters, more specifically the mass loadings on the FIGAERO filter (*m*_filter_). The second highest importance is attributed to SWD. In contrast, the MLR indicates a negligible contribution of these two parameters, and attributes parameters related to temperature, i.e., *T*_ambient_, *T*_room_, and its difference Δ*T*_room‑ambient_, the highest importance. This discrepancy between the most important parameters between the ML models and the MLR might be related to nonlinear relations between *T*_max_ and both the mass loading on the FIGAERO filter and SWD, which are not captured by MLR, only by the ML models. The high importance given by MLR to temperature-related parameters could be caused by the overall seasonal pattern with colder temperatures in winter and warmer in summer. This annual cycle is identified as an overall trend in the data, which is why MLR associated a high importance to it, but the ML models learned that temperature alone is not driving the changes in *T*_max_ and that the mass loading on the FIGAERO filter is the more dominant parameter. In addition, the better model performance of RF and XGBoost regressor compared to MLR (as mentioned above) suggests that the results from the ML models explain the underling relations between *T*_max_ and the 18 parameters better than MLR.

While Figure 3b provides the overall importance according to SHAP, Figure 4 presents a more detailed view on the SHAP values, as it shows whether lower or higher values of the different parameters lead to an increased or decreased prediction of *T*_max_ for RF (Figure 4a) and the XGBoost Regressor (Figure 4b). The parameters are sorted by their overall importance from top to bottom, corresponding to Figure 3b. Figure 4 can be interpreted as follows: positive SHAP values indicate that this parameter increases the predicted value of *T*_max_, while negative SHAP values indicate this parameter decreases the predicted value of *T*_max_. This effect depends on whether the parameter value is low or high, as indicated by the blue to red color scale for individual model predictions (dots).

Of the four different parameter groups, the FIGAERO parameters (especially mass deposited on the filter (m_filter_)) were identified as the most important for *T*_max_ variation. With the mass deposited on the FIGAERO filter playing an important role, it is unsurprising that the mass loading of levoglucosan (*m*_I(C6H10O5)_) is also among the more important parameters in the FIGAERO parameter group. Both ML models associate lower mass loadings on the FIGAERO filter with higher *T*_max_ values. This can be connected to the influence of the background signal at low mass loadings, which appears at higher *T*_max_ values (as discussed earlier in Section 3.1). We speculate that the observed trend of higher *T*_max_ at lower mass loadings is specific to the clean environment of the Arctic and resulting low *m*_filter_, leading to increased particle-filter interactions or relatively enhanced contributions from a generally low Arctic background signal. The importance of m_filter_ also holds when excluding thermograms in Category 6 (Figure 2) or applying thermogram background corrections (see Supporting Information Sections S10 and S11). Extended analyses on thermograms from two other organic compounds measured with the FIGAERO–CIMS further support the importance of the mass on the FIGAERO filter with higher *T*_max_ at lower mass loadings (Supporting Information Section S12). The ratios *m*_I(C6H10O5)‑:_ *m*_filter_ and *m*_I(C6H10O5)‑_:ORG are both identified as being of low importance by all three models (Figure 3a,b). These ratios can indicate the mixing ratio of the particles. Overall, both ratios are elevated during the winter months (Supporting Information Figure S5), most likely reflecting a higher contribution of biomass burning emissions transported from lower latitudes to the Arctic. Consequently, as mentioned in Section 3.1, it is reasonable to assume that the particles are internally mixed when reaching the Arctic, thus, the mixing state of the particles is of only minor importance.

Of the meteorological parameters, *T*_ambient_ and SWD are on average most relevant, that is, they have the highest influence on *T*_max_. However, the MLR gives a high importance to *T*_ambient_ and a lower one to SWD, while the RF and XGBoost Regressor indicate the opposite for both evaluation parameters (Figure 3a,b). As there is a positive linear correlation between *T*_ambient_ and SWD (*r* = 0.42, Supporting Information Figure S1) due to the pronounced seasonality of solar radiation and temperature at the measurement site with its location north of the Arctic circle,^69^ it can be expected that MLR assigns a similar importance to both parameters. However, the MLR might assign a higher importance to *T*_ambient_, as it fluctuates less throughout the year compared to SWD (Supporting Information Figure S5). Figure 4 reveals that higher *T*_ambient_ values are related to lower *T*_max_ values in both the RF regressor (Figure 4a) and the XGBoost regressor (Figure 4b). A similar tendency can be observed for the SWD. This means that at warmer and sunlit ambient conditions (i.e., in summer), levoglucosan thermograms exhibit lower *T*_max_ (and higher apparent volatility) compared to colder and darker conditions in winter. RH, WD, and the time the air mass spent over the land before it arrived at the measurement site have negligible relevance for *T*_max_ in all three models and in both performance parameters (Figure 3a,b). As the WD during NASCENT was mainly from two directions,^44^ it is reasonable that the WD is less relevant for *T*_max_.

The physical parameter group is on average the group with the least importance (Figure 3). The largest discrepancy among the models is observed for the importance of PNC_0.01–2.5_, and *D*_mean_, where the MLR gives a much lower importance to these parameters than the ML Regressors. All models show that PM_0.18–2.5_ and *D*_peak_ play a minor role for *T*_max_. This suggests that the particle number and their average size could influence *T*_max_, at least partly. From the SHAP summary plot (Figure 4a,b) there appears to be a tendency to higher *T*_max_ with fewer particles. If the particles are not only fewer in number but also smaller in size, then this observed relation to higher *T*_max_ could point to very clean conditions, which were close to the background and exhibited a large signal-to-noise ratio and would resemble the thermograms classified in Category 6 in Figure 2. Figure 4 also shows that larger particles (*D*_mean_) are related to higher *T*_max_ values, which supports the study by Ylisirniö et al.,^39^ which observed slightly higher *T*_max_ for particles of 300 nm compared to the smaller 80 nm particles (at similar mass loadings). This effect was attributed to differences in surface-to-volume ratios: larger particles, with smaller surface-to-volume ratios, exhibit lower desorption rates as their smaller relative surface area requires more time to evaporate compared to smaller particles.^39^

The three parameters in the group of chemical properties appear on average to be more important than the physical parameters but less important than the FIGAERO parameters (Figure 3). Especially the O:C ratio is shown to be important, where higher O:C ratios are associated with lower *T*_max_ for both ML regressors (Figure 4). This could be related to particle matrix effects, for example, by modifying intermolecular interactions and particle phase state. As the MLR indicates nonlinearities in the data, this result could also point to a more complex relation between the O:C ratio and some of the other as important identified parameters, such as the mass loadings on the FIGAERO filter and *T*_ambient_ or SWD. Gramlich et al.^47^ reported higher O:C ratios at the measurement site during the summer due to the dominating marine source of methanesulfonic acid, which exhibits a high O:C ratio. The fraction of organics (ORG:PM_0.18–2.5_) shows higher importance in the ML models compared to the MLR, where, based on SHAP (Figure 4), lower ratios are related to higher *T*_max_ values or more noisy thermograms. As the mass loading is the dominating impact parameter, with higher mass loadings in winter compared to summer (Supporting Information Figure S5), the impact of the physicochemical differences between the two seasons appears to be a secondary effect. Additional analysis on including inorganic species, which provide another source of aerosol particles in the Arctic, shows that these species have a negligible effect on *T*_max_ (Supporting Information Section S13).

### 3.3 Relation of *T*_max_ and FIGAERO Mass Loading

As shown in Section 3.2, the mass loading on the FIGAERO filter has the largest impact on *T*_max_ and SWD has the second largest impact. To investigate these relations in more detail, Figure 5 shows *T*_max_ as a function of the mass collected on the FIGAERO filter, color coded by the SWD. Overall, despite the largest relevance of the mass loadings on the FIGAERO filter of all 18 parameters on *T*_max_, there appears to be no clear linear relation between the mass loading and *T*_max_. However, at lower mass loadings, *T*_max_ spans a wide range including the highest *T*_max_ values, whereas at higher mass loadings, *T*_max_ is limited to consistently lower temperatures, and at higher SWD, *T*_max_ tends to be lower. This relation is also in line with the predictions of the RF and XGBoost regressors discussed in Section 3.2 and still holds when considering also those thermograms that had to be excluded from the analysis due to missing values in the other parameters (see Supporting Information Figure S24).

**Figure 5:**
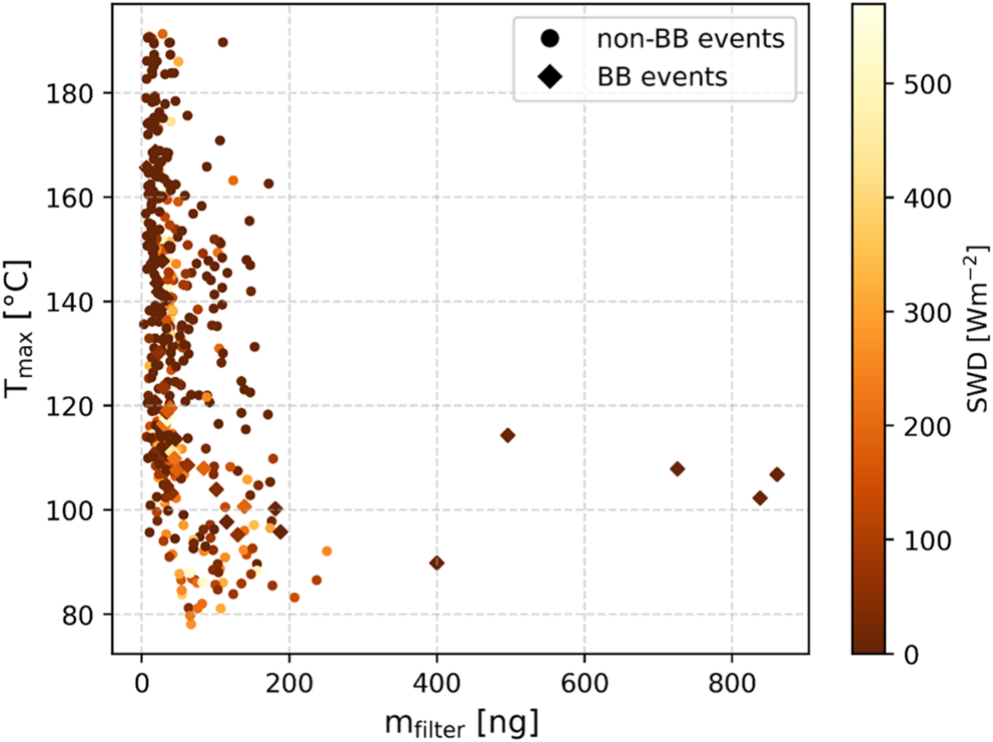
*T*_max_ as a function of m_filter_, color coded by SWD. The data corresponding to non-BB events are presented in circles, whereas the BB events are shown as diamonds.

As mentioned in Section 3.2, the importance of SWD for *T*_max_ suggests that there is also a seasonal effect, with low SWD dominating in the dark winter months and high SWD in the bright summer months. Figure 5 shows that there is a tendency that lower mass loadings dominate at higher SWD, and higher mass loadings dominate at lower SWD, except for the BB events, which occur throughout both seasons. As the mass loadings are a result of atmospheric concentrations, sampling duration, and sampling volume, the mass loadings can directly be related to atmospheric concentrations, if the sampling duration and volume are kept similar. Hence, lower mass loadings reflect lower ambient mass concentrations and vice versa. Lower mass concentrations in the sunlit part of the year and higher concentrations in the darker months are expected from the typical annual cycle at our measurement site.^70^

Higher mass loadings occur independently of BB events; therefore, the BB events should not have driven the ML models. Additionally, the relation presented in Figure 5 also indicates why MLR attributed only a negligible influence of *m*_filter_ on *T*_max_, as this relationship is clearly not linear. Only few previous studies have addressed the impact of the mass loading on the FIGAERO filter on *T*_max_. While Huang et al.^38^ found an increase in *T*_max_ with higher mass loadings (for mass loadings between approximately 200 ng and 2 μg), reaching a plateau at around 2–4 μg on the filter, Ylisirniö et al.^39^ report no such an effect. Cai et al.^71^ report similar tendencies for changes in *T*_max_ (5 to 15 °C for 35 to 57% of all compounds) as Huang et al.^38^ but considered slightly higher mass loadings (1.2 to 15 μg). However, the mass loadings in Ylisirniö et al.^39^ were in the range between 100 and 200 ng, lower than the lower range considered in Huang et al.^38^ and also lower than the mass loadings in Cai et al.^71^ Further, for the lower mass loadings reported by Huang et al.,^38^ a large uncertainty in *T*_max_ is reported. Our mass loadings are similar to Ylisirniö et al.^39^ and in the lower range of Huang et al.^38^ Accordingly, *T*_max_ shows substantial variability with no pronounced dependence on the mass loading on the FIGAERO filter, although a tendency to lower *T*_max_ at higher mass loadings is observed, which is in line with the model predictions discussed in Section 3.2. This suggests that the effect of the mass loading on *T*_max_ might be only relevant from a certain threshold, which starts in the range between 200 ng and 2 μg. Given that at low mass loadings the signal-to-noise ratio is much lower, the reliability of *T*_max_ at low mass loadings is overall more uncertain than at higher mass loadings. This further suggests that the direction in which a shift of *T*_max_ is observed at increasing mass loadings depends on the starting conditions: If the mass loadings on the filter are low (i.e., when the signal approaches background conditions), *T*_max_ decreases with increasing mass loadings, as the influence from the background decreases. If, however, the mass loadings on the filter are high, *T*_max_ increases with increasing mass loadings as the particles are exposed to diffusion or heat transfer limitations on the filter.

Our results show that among the 18 different parameters investigated, the mass loading on the FIGAERO filter has the largest influence on levoglucosan *T*_max_, with a tendency of overall lower *T*_max_ at higher mass loadings. Overall, higher mass loadings were associated at times with lower SWD, which refers to the winter half of the year, while lower mass loadings were observed at higher SWD in the summer, suggesting a driving effect from the mass loading with an additional seasonal effect, due to the location of the measurement site far north of the Arctic circle. At low mass loadings, the signal-to-noise ratio is much lower; hence, the reliability of *T*_max_ at low mass loadings is overall more uncertain than at higher mass loadings. Therefore, *T*_max_ shifts to lower values (and increased volatility) when the mass loading is increased from levels near the background, while *T*_max_ increases (and the volatility decreases) when the mass loadings are decreased further toward the background conditions. The relevance of the mass on the FIGAERO filter for variations in *T*_max_ implies that controlling this parameter can help reduce the variability in *T*_max_ and therefore improve the *T*_max_-derived volatility. Based on our results, mass loadings of around 150 ng are recommended. We recommend future dedicated laboratory experiments to investigate mass loadings for stable *T*_max_ in more detail. This could be achieved by depositing a series of known amounts of material on the FIGAERO filter, e.g., starting from 150 ng up to 1 μg, in 100 ng steps. Hence, the mass loading should be considered in the analysis and interpretation of FIGAERO–CIMS thermograms. This is particularly of importance when *T*_max_ is used to derive volatility and partitioning of semivolatile species between the gas and condensed phase. Ideally mass loadings with the FIGAERO should be kept constant to minimize their impact on *T*_max_ and thus on the derived volatility. To keep the mass loadings on the filter stable during measurements, the FIGAERO–CIMS user could use the real-time mass concentrations from instruments usually deployed aside, such as ACSM/AMS or DMPS/SMPS, to adapt the sampling period in real time. More broadly, we demonstrate the value of machine learning approaches for characterizing instrumental backgrounds in complex ambient or laboratory data. Such approaches can reveal subtle patterns and dependencies that are difficult to obtain otherwise.

## Supplementary Material



## Notes

Calculation of FIGAERO–CIMS parameters (S1); definition of BB and non-BB thermograms (S2); overview of parameters used (Table S1) and their respective values during non-BB and BB times (Table S2); correlation matrix of all parameters (Figure S1); comparison thermogram with high and low noise (Figure S2); time series signal-to-noise ratio (Figure S3); blank thermograms (Figure S4); time series of parameters (Figure S5); model results (Figure S6–S8); importance of parameters on *T*_max_ without downweighing (Figures S9 and S10); impact of 18 parameters on *T*_max_ without thermograms in category 6 (S10); impact of 18 parameters on *T*_max_ applying background correction (S11); impact of 18 parameters on *T*_max_ for other organic compounds than levoglucosan (S12); influence of inorganic components on *T*_max_ (S13); and relation *T*_max_ and FIGAERO mass loading with extended data (Figure S24) (PDF)

## ⊥

Karolina Siegel: Swedish Meteorological and Hydrological Institute (SMHI), Norrköping, 60176, Sweden
