# Supplementary material for: A Machine Learning Approach to Understand Thermal Desorption Profiles of Levoglucosan from FIGAERO–CIMS
Source: Environ Sci Technol. 2026 May 20;60(21):15101–12. doi: 10.1021/acs.est.5c18488 (PMC13235551; doi:10.1021/acs.est.5c18488)
Supplement: Supplementary file 1 [file es5c18488_si_001.pdf]

Supporting Information for

A Machine Learning Approach to Understand Thermal Desorption Profiles of Levoglucosan from FIGAERO-CIMS

Yvette Gramlich[1, ‡], Roman Spahr[2, ‡], Abhishek Upadhyay[1], Karolina Siegel[3, §], Sophie L. Haslett[3], Radovan Krejci[3], Karl Espen Yttri[4], Claudia Mohr[1,2,]*

[1]PSI Center for Energy and Environmental Science, 5232 Villigen PSI, Switzerland

[2]Department of Environmental Systems Science, ETH Zurich, 8092 Zurich, Switzerland

[3]Department of Environmental Science, Stockholm University, Stockholm 11418, Sweden

[4]NILU, Kjeller 2027, Norway

*Corresponding author: claudia.mohr@psi.ch

Content of Supporting Information:

Number of pages: 32

Number of figures: 24

Number of tables: 2

Sections included:

S1 Calculation of FIGAERO-CIMS Parameters S4
S2 Definition of BB and non-BB Thermograms S5
S3 Overview Parameters Used S6
S4 Correlation of the Parameters S8
S5 Thermogram Noise S9
S6 Thermogram Blanks S10
S7 Time Series of Parameters S11
S8 Model Outputs S12
S9 Comparison to Using T_{max} Without Downweighing S14
S10 Impact of 18 Parameters on T_{max} without Thermograms in Category 6 .... S16
S11 Impact of 18 Parameters on T_{max} Applying Background Correction S18
S12 Impact of 18 Parameters on T_{max} for other Organic Compounds than Levoglucosan S21
S13 Influence of Inorganic Components on T_{max} S27
S14 Relation of T_{max} and FIGAERO Mass Loading S30

Figures included:
Figure S1. Correlation matrix S8
Figure S2. Example of thermograms with different noise. S9
Figure S3. Time series of the signal-to-noise ratio of levoglucosan S9
Figure S4. Levoglucosan blank thermograms S10
Figure S5. Time series of parameters S11
Figure S6. Multiple Linear Regression (MLR) coefficients for the 18 different parameters using T_{max} as target value. S12
Figure S7. Model output for RF using downweighed T_{max} as target value. S12
Figure S8. Model output for XGBoost using downweighed T_{max} as target value. S13
Figure S9. Comparison of the normalized importance of parameters for T_{max} variation, grouped into the categories Meteorological Parameters, Physical Parameters, Chemical Parameters, FIGAERO Parameters, retrieved from the three different models, when no downweighing is applied to T_{max} S14
Figure S10. SHAP summary plot when no downweighing is applied to T_{max} S15
Figure S11. Comparison of the normalized importance of parameters for T_{max} variation, grouped into the categories Meteorological Parameters, Physical Parameters, Chemical Parameters, FIGAERO Parameters, retrieved from the three different models, when data from category 6 (Figure 2) is excluded. S16
Figure S12. SHAP summary plot, when data from category 6 (Figure 2) is excluded S17
Figure S13. Example of blank-corrected thermograms. S18
Figure S14. Comparison of the normalized importance of parameters for T_{max} variation, grouped into the categories Meteorological Parameters, Physical Parameters, Chemical Parameters,

FIGAERO Parameters, retrieved from the three different models, when using blank-corrected thermograms. S19
Figure S15. SHAP summary plot, when using blank-corrected thermograms S20
Figure S16. Overview of $I(C_3H_4O_4)^-$ thermograms S22
Figure S17. Overview of $I(C_9H_{14}O_5)^-$ thermograms S22
Figure S18. Comparison of the normalized importance of parameters for T_{max} variation, grouped into the categories Meteorological Parameters, Physical Parameters, Chemical Parameters, FIGAERO Parameters, retrieved from the three different models, when using $I(C_3H_4O_4)^-$ thermograms. S23
Figure S19. SHAP summary plot for (a) RF Regressor and (b) XGBoost Regressor for T_{max} of $I(C_3H_4O_4)^-$. S24
Figure S20. Comparison of the normalized importance of parameters for T_{max} variation, grouped into the categories Meteorological Parameters, Physical Parameters, Chemical Parameters, FIGAERO Parameters, retrieved from the three different models, when using $I(C_9H_{14}O_5)^-$ thermograms. S25
Figure S21. SHAP summary plot for (a) RF Regressor and (b) XGBoost Regressor for T_{max} of $I(C_9H_{14}O_5)^-$. S26
Figure S22. Comparison of the normalized importance of parameters for T_{max} variation, grouped into the categories Meteorological Parameters, Physical Parameters, Chemical Parameters, FIGAERO Parameters, retrieved from the three different models, when an inorganic species is included as additional parameter (sodium: Na^+). S28
Figure S23. SHAP summary plot for (a) RF Regressor and (b) XGBoost Regressor including Na^+ as a parameter. S29
Figure S24. T_{max} as function of m_{filter}, color coded by SWD. S30

Tables included:

Table S1: Overview of parameters investigated for influence on T_{max}, organized by the four categories: 'Meteorological parameters', 'Physical parameters', 'Chemical parameters', and 'FIGAERO parameters' (instrumental parameters). S6
Table S2: Overview of average and standard deviation for all 18 parameters during non-BB and BB event times. S7

S1 Calculation of FIGAERO-CIMS Parameters

The mass loadings of the FIGAERO-CIMS filter (m_{filter}) were derived from mass concentrations calculated using Equation (S1)[1] multiplied by the sampled volume of air (Equation (S2)):

$$conc_i = \frac{F*10^{-12}*m_{mol}*L_{in}*MW_i*10^9}{cal*h_{time}*part_{flow}*coll_{time}} \quad (S1)$$

$$m_{filter} = \frac{part_{flow}*coll_{time}*\sum_i^n conc_i}{10^9} \quad (S2)$$

with $conc_i$ as the mass concentration of one ion i (e.g., $I(C_6H_{10}O_5)^-$) in µg m^{-3}, F the FIGAERO-CIMS signal in ion counts, m_{mol} the molar concentration in mol L^{-1}, L_{in} the total flow in L going into the ion molecule reaction chamber (IMR), MW_i the molecular weight in g mol^{-1}, cal the sensitivity of 22 counts s^{-1} ppt^{-1} [2], h_{time} the duration of desorption in seconds, $part_{flow}$ the particle sampling flow in LPM during the particle collection period, $coll_{time}$ the particle collection duration in minutes, n the number of compounds measured with the FIGAERO-CIMS.

S2 Definition of BB and non-BB Thermograms

The thermograms used were classified as biomass burning (BB) thermograms and non-BB thermograms according to the definition of BB events reported in our previous study in Gramlich et al.[1]. In brief, the time series of levoglucosan and equivalent Black Carbon (eBC) were utilized, and the identification of BB events was approached in two steps. First, the peak of the BB plume was identified as extreme value when both compounds reached the 97th percentile, calculated in several ways (for the full month, and using running windows over around 15, 30 and 45 days). Second, the start and end times were identified by the points around the peak where changes between consecutive time points approached or fluctuated around zero. Both steps were done for both compounds and only overlapping time periods were taken. In total 7 BB event periods were identified during 2020. For more details on the identification of the BB periods the reader is referred to Gramlich et al.[1]. In the current work the thermograms of BB events included in the analysis were filtered based on the availability of data from all other 18 parameters.

S3 Overview Parameters Used

Table S1: Overview of parameters investigated for influence on T_{max}, organized by the four categories: 'Meteorological parameters', 'Physical parameters', 'Chemical parameters', and 'FIGAERO parameters' (instrumental parameters).

Parameter [unit]	Abbreviation	Measurement location (instrument)	Time resolution
**Meteorological Parameters**			
Ambient Temperature [°C]	$T_{ambient}$	Zeppelin Observatory (Vaisala)	1 h
Relative Humidity [%]	RH	Zeppelin Observatory (Vaisala)	1 h
Wind Direction [°]	WD	Zeppelin Observatory (Vaisala)	1 h
Short-Wave Downward (global) Radiation [W m^{-2}]	SWD	BSRN Station Ny-Ålesund (Pyranometer)	1 min
Land Count	land count	trajectories from Freitas et al.[3]	1 d
**Physical Parameters**			
Particle Number Concentration (D = 0.01−0.8 µm) [cm^{-3}]	$PNC_{0.01-0.8}$	Zeppelin Observatory (DMPS)	1 h
Particle Mass Concentration (D = 0.18−2.5 µm) [µg m^{-3}]	$PM_{0.18-2.5}$	Zeppelin Observatory (FIDAS)	1 h
Particle Mean Diameter (D = 0.01−0.8 µm) [nm]	D_{mean}	Zeppelin Observatory (DMPS)	1 h
Particle Peak Diameter (D = 0.01−0.8 µm) [nm]	D_{peak}	Zeppelin Observatory (DMPS)	1 h
**Chemical Parameters**			
Organic Mass Concentration (D = 0.11−3.5 µm) [µg m^{-3}]	ORG	Zeppelin Observatory (ToF-ACSM)	1 h
Ratio organic mass concentration to $PM_{0.18-2.5}$	ORG: $PM_{0.18-2.5}$	-	-
O:C of organics	O:C	Zeppelin Observatory (FIGAERO)	1 h
**FIGAERO Parameters**			
Total Mass Loading on filter [ng]	m_{filter}	Zeppelin Observatory (FIGAERO)	2.5 h
$I(C_6H_{10}O_5)^-$ Mass Loading [ng]	$m_{I(C_6H_{10}O_5)^-}$	Zeppelin Observatory (FIGAERO)	2.5 h
Ratio $I(C_6H_{10}O_5)^-$ Mass Loading to total Mass Loading on Filter	$m_{I(C_6H_{10}O_5)^-}$:m_{filter}	-	-
Ratio $I(C_6H_{10}IO_5)^-$ Mass Concentration to Organic Mass Concentration	$PM_{I(C_6H_{10}O_5)^-}$: ORG	-	-
Room Temperature [°C]	T_{room}	Zeppelin Observatory (FIGAERO)	2.5 h
Difference Room Temperature to Ambient Temperature [°C]	$\Delta T_{room-ambient}$	-	-

Table S2: Overview of average (Mean) and standard deviation (Std) for all 18 parameters during non-BB and BB event times.

Parameter	Non-BB events		BB events	
	Mean	Std	Mean	Std
$T_{ambient}$ [°C]	-7.9	8.0	-0.8	5.3
RH [%]	82.2	12.3	76.5	15.2
WD [°]	161.3	85.3	153.7	18.9
SWD [W m^{-2}]	90.7	132.5	76.5	127.1
Land count [%]	17.4	10.5	27.7	17.6
$PNC_{0.01-0.8}$ [cm^{-3}]	158.1	152.2	146.9	123.4
$PM_{0.18-2.5}$ [µg m^{-3}]	1.7	2.5	2.2	3.6
D_{mean} [nm]	126.3	49.6	114.6	38.8
D_{peak} [nm]	120.7	70.5	97.3	54.4
ORG [µg m^{-3}]	0.1	0.1	0.2	0.3
ORG:$PM_{0.18-2.5}$ [%]	12.8	12.8	15.7	15.4
O:C	1.0	0.5	0.9	0.4
m_{filter} [ng]	51.5	43.2	135.9	227.0
$m_{I(C6H10O5)-}$ [ng]	0.2	0.3	1.7	3.3
$m_{I(C6H10O5)-}$: m_{filter} [%]	0.4	0.4	0.8	0.8
$PM_{I(C6H10O5)-}$:ORG [%]	0.5	0.9	0.9	0.9
T_{room} [°C]	28.3	4.6	27.7	5.5
$\Delta T_{room-ambient}$ [°C]	36.2	7.5	28.4	5.6

S4 Correlation of the Parameters

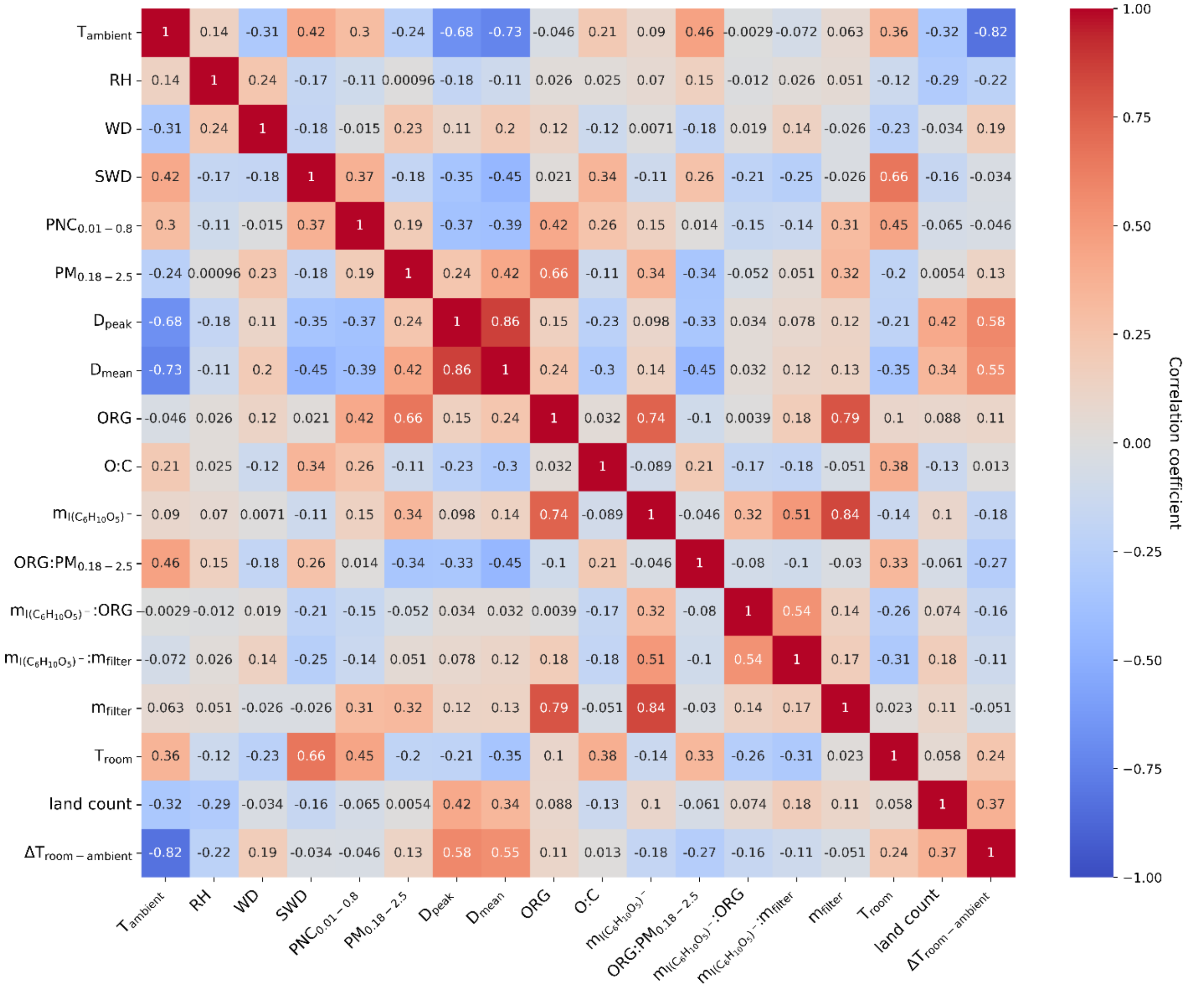

Figure S1. Correlation matrix of the time series of all parameters considered in the analysis, resampled to the time resolution of the FIGAERO-CIMS. The numbers in the matrix represent the Pearson correlation coefficient, where values between 0 and 1 indicate a positive linear correlation, and values between 0 and -1 a negative linear correlation.

S5 Thermogram Noise

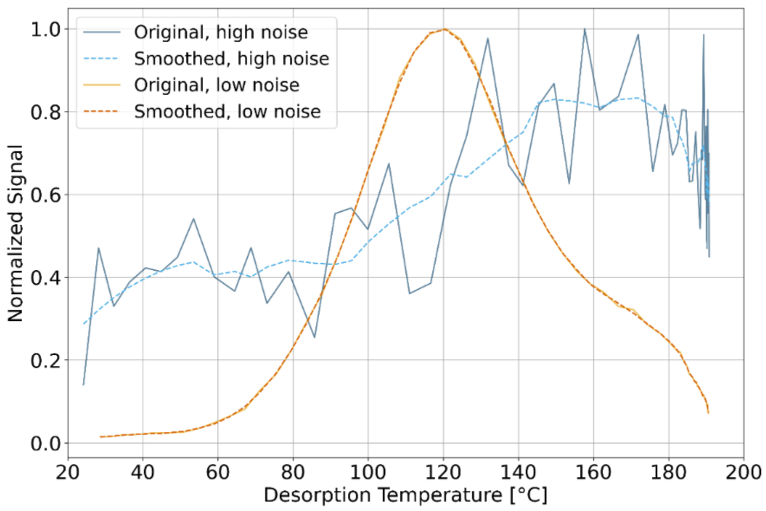

Figure S2. Example of thermograms with different noise – a high noise (dark blue, 8 % average deviation between the original and the smoothed curve) and a thermogram with low noise (yellow, 0.3 % average deviation between the original and the smoothed curve). The dashed lines represent the smoothed versions applying a Savitzky-Golay filter[4] (light blue: smoothed high noise thermogram, orange: smoothed low noise thermogram).

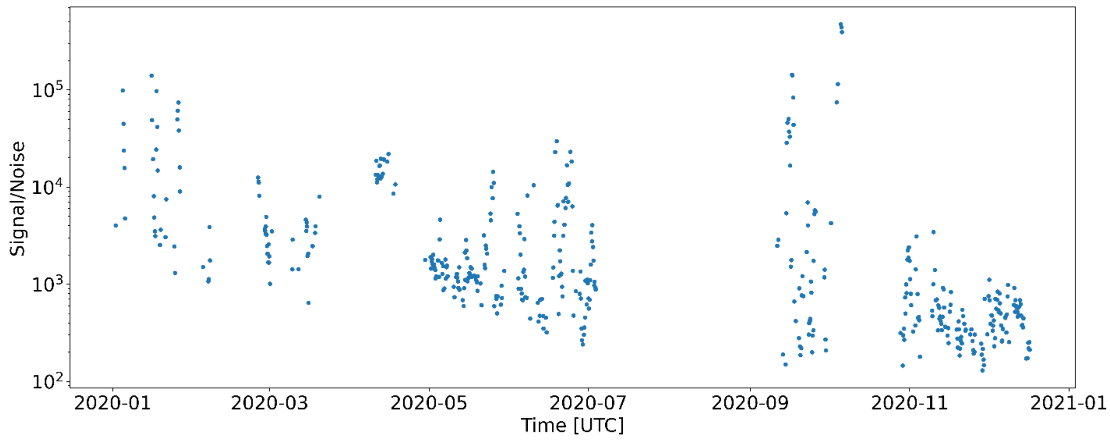

Figure S3. Time series of the signal-to-noise ratio of levoglucosan over the entire measurement period. Here the signal is the absolute signal at T_{max} and the noise is the average deviation between the smoothed and the original thermogram.

S6 Thermogram Blanks

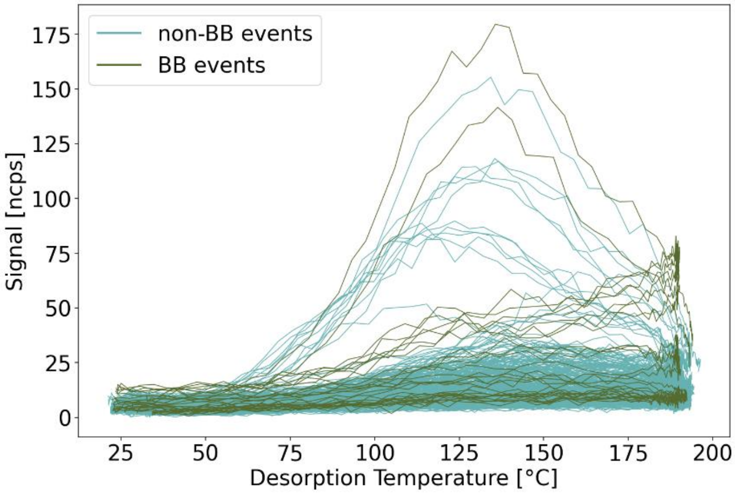

Figure S4. Levoglucosan blank thermograms. The color code of the thermograms indicates whether it refers to a blank measured during a BB event or a blank during non-BB event time. The blanks were obtained by placing a second FIGAERO filter upstream the FIGAERO sampling filter as described in Gramlich et al.[5]. Every third cycle of the FIGAERO throughout the measurement period was a blank.

S7 Time Series of Parameters

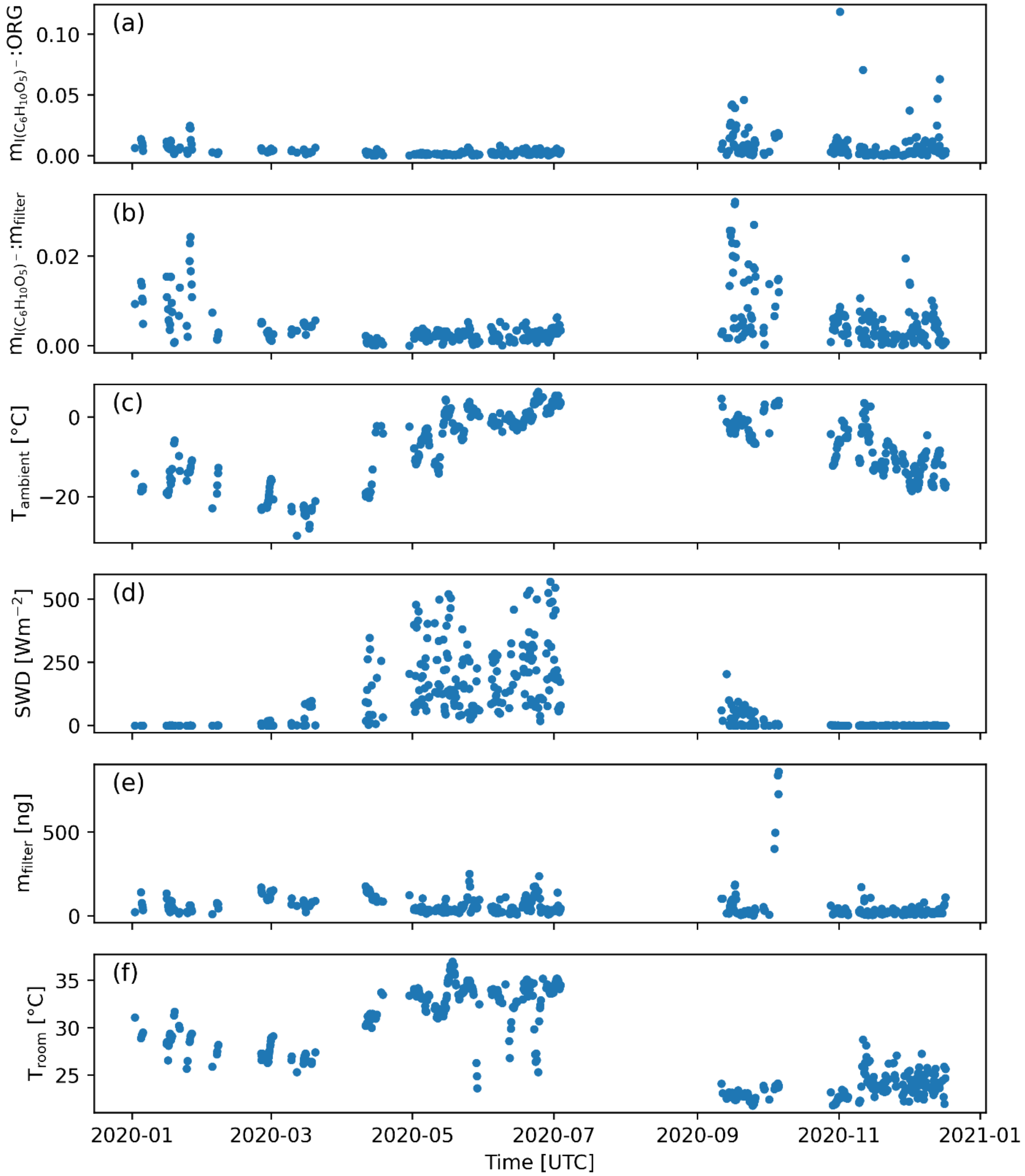

Figure S5. Time series of parameters (a) $m_{I(C6H10O5)-:ORG}$ (b) $m_{I(C6H10O5)-:}m_{filter}$ (c) $T_{ambient}$ (d) SWD and (e) m_{filter} (f) T_{room}.

S8 Model Outputs

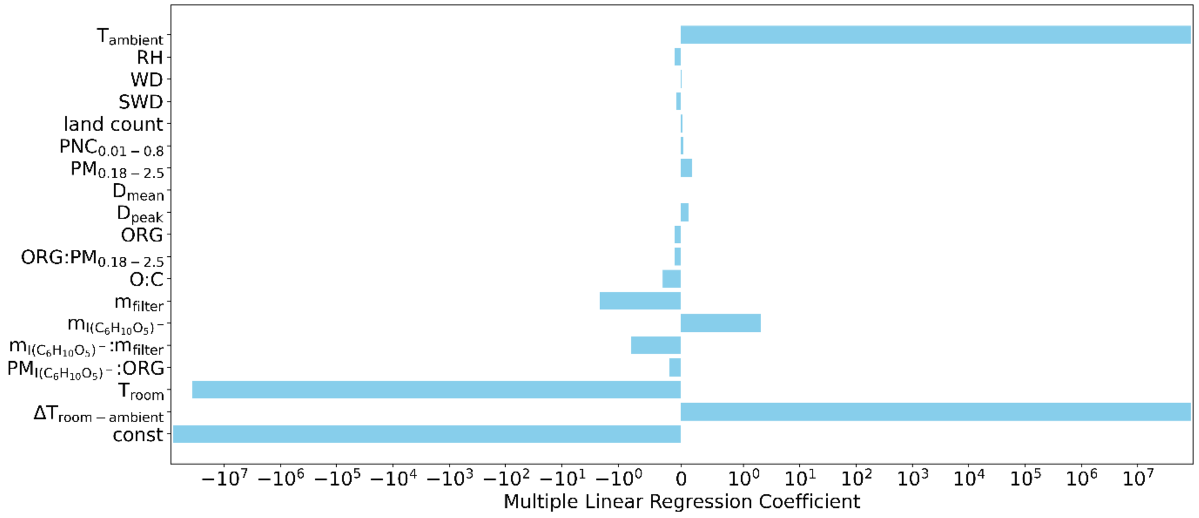

Figure S6. Multiple Linear Regression (MLR) coefficients for the 18 different parameters using T_{max} as target value.

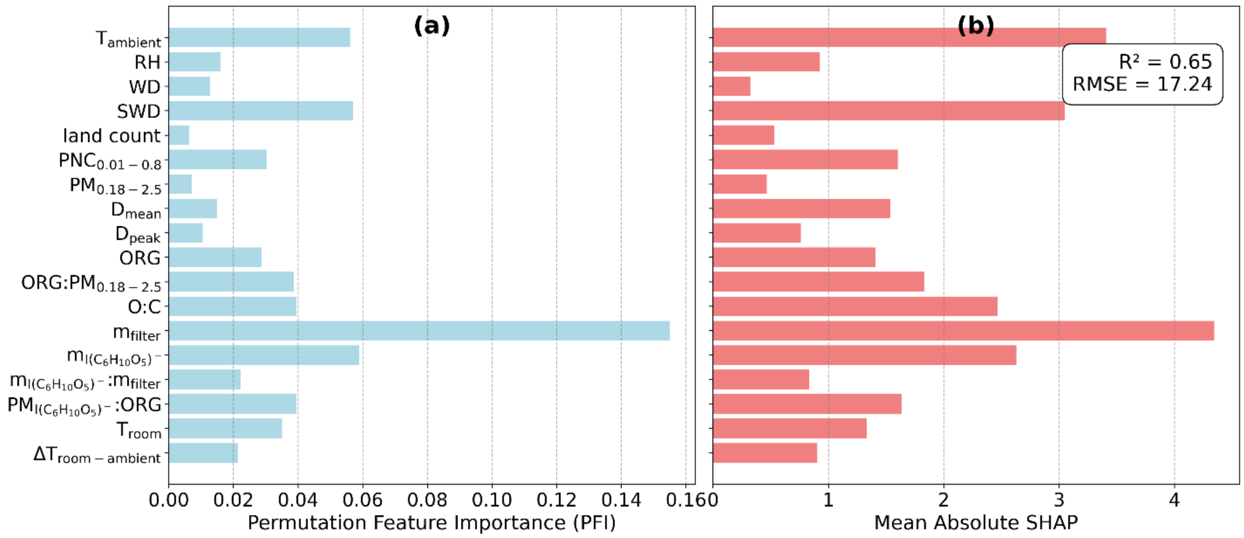

Figure S7. Model output for RF using downweighed T_{max} as target value. (a) Permutation Feature Importance (PFI) and (b) Shapley Additive explanations (SHAP).

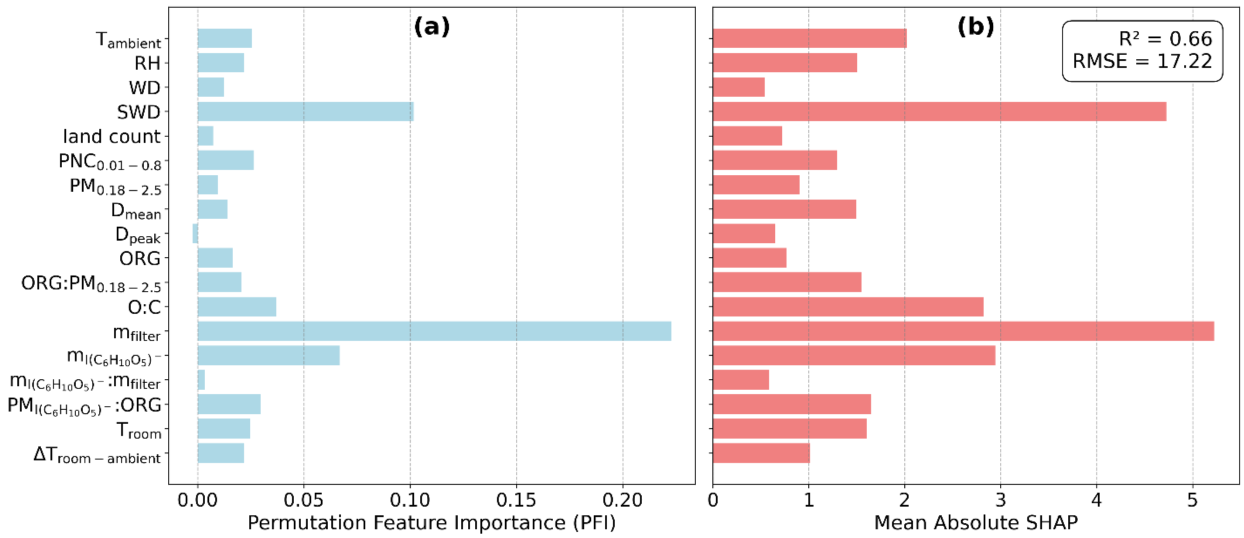

Figure S8. Model output for XGBoost using downweighed T_{max} as target value. (a) Permutation Feature Importance (PFI) and (b) Shapley Additive explanations (SHAP).

S9 Comparison to Using T_{max} Without Downweighing

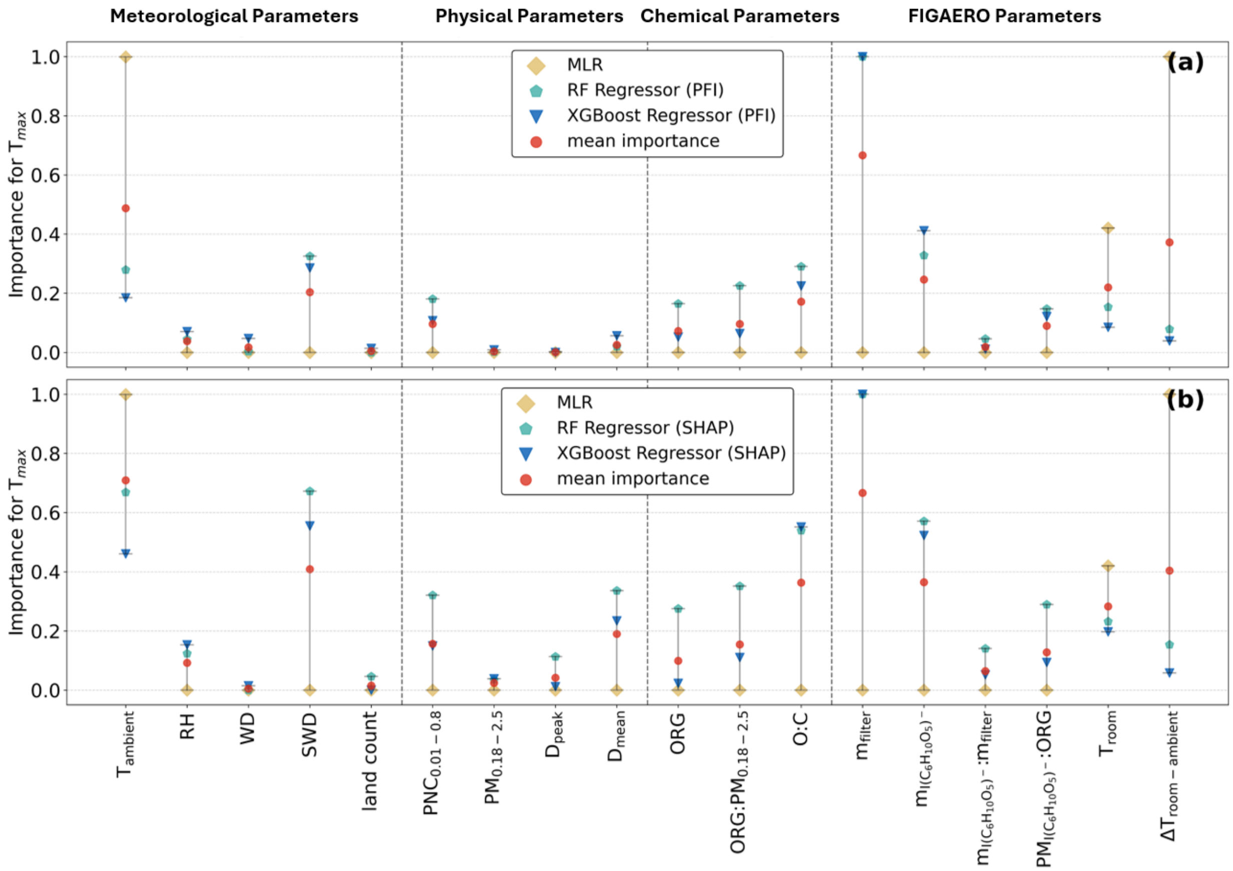

Figure S9. Comparison of the normalized importance of parameters for T_{max} variation, grouped into the categories Meteorological Parameters, Physical Parameters, Chemical Parameters, FIGAERO Parameters, retrieved from the three different models, when no downweighing is applied to T_{max}. To make the models comparable, the importances of the parameters per model were normalized using the MinMaxScaler from the scikit-learn library[6]. (a) shows the normalized importance of the permutation feature importance (PFI), while (b) shows the normalized importance of the SHAP values. In (a) and (b) the MLR values refer to the normalized MLR regression coefficients. The mean importance indicates the average over all the models, for each parameter, respectively.

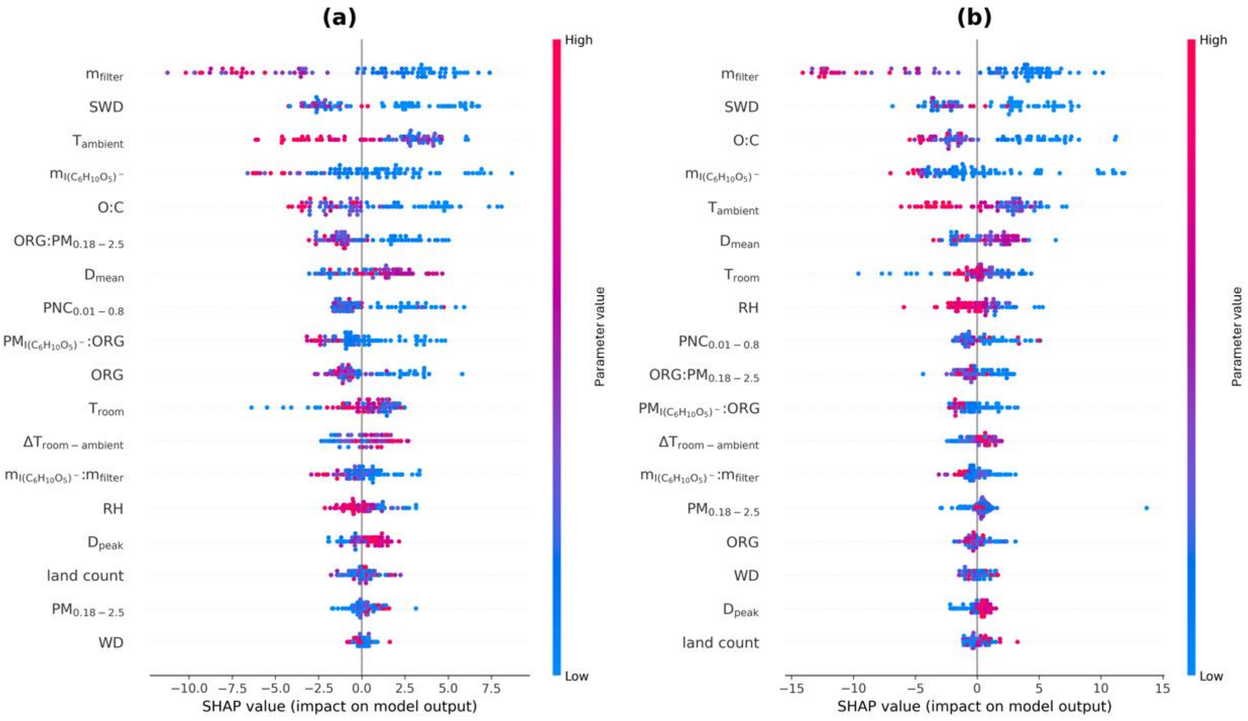

Figure S10. SHAP summary plot when no downweighing is applied to T_{max} for (a) RF Regressor and (b) XGBoost Regressor. From top to bottom the parameters are sorted according to their overall importance according to SHAP, while the colored dots indicate the direction in which a high (red) or a low (blue) parameter value are related to T_{max}. The dots represent each one prediction of T_{max}. Negative values on the x-axis indicate a decrease in T_{max}, while positive values on the x-axis indicate an increase in T_{max}.

S10 Impact of 18 Parameters on T_{max} without Thermograms in Category 6

Figure S11 shows the impact of the 18 parameters on T_{max} when Category 6 thermograms (in total 40 thermograms) were excluded from the analysis, and Figure S12 the corresponding SHAP summary plot. The most important factor remains the mass on the FIGAERO filter, similar to the base case presented in the main manuscript.

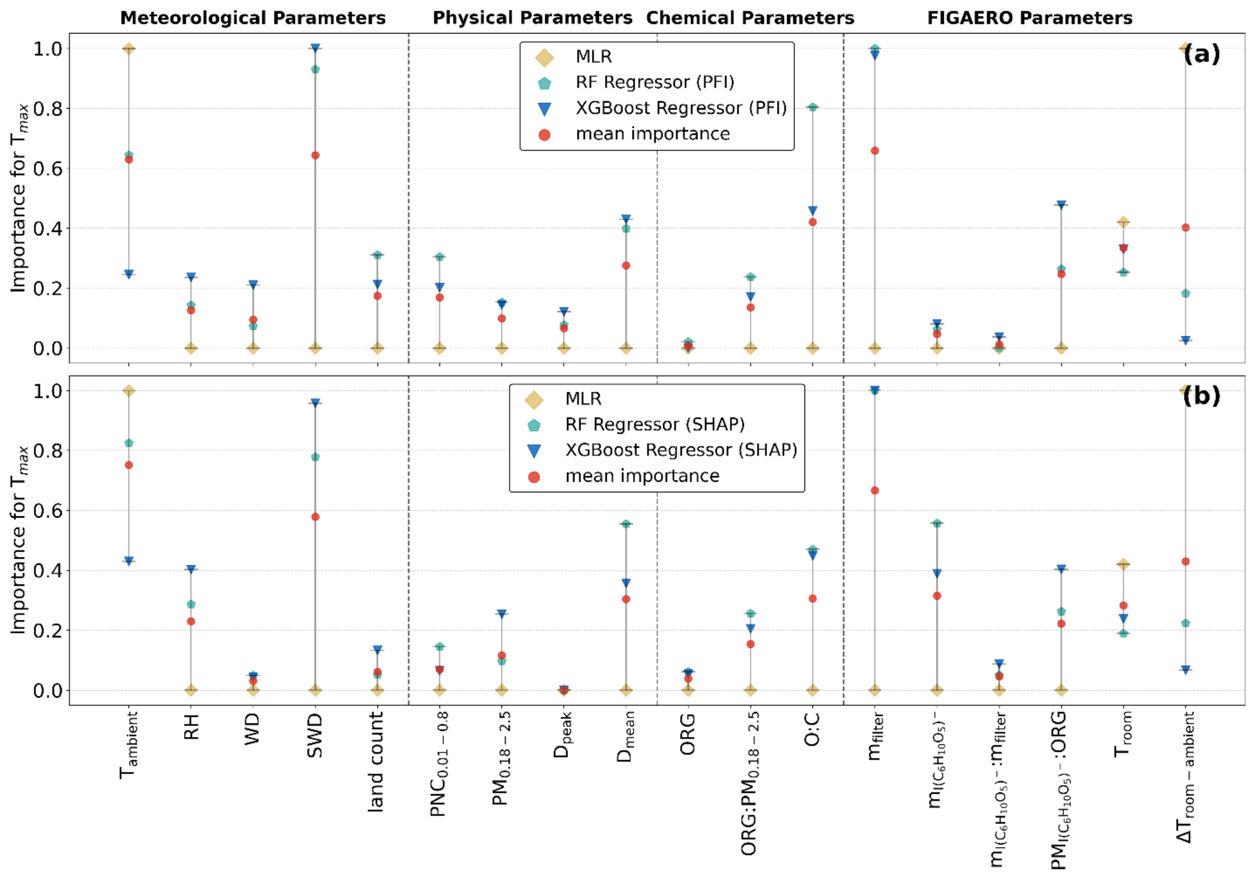

Figure S11. Comparison of the normalized importance of parameters for T_{max} variation, grouped into the categories Meteorological Parameters, Physical Parameters, Chemical Parameters, FIGAERO Parameters, retrieved from the three different models, when data from Category 6 (Figure 2) is excluded. To make the models comparable, the importances of the parameters per model were normalized using the MinMaxScaler from the scikit-learn library[6]. (a) shows the normalized importance of the permutation feature importance (PFI), while (b) shows the

normalized importance of the SHAP values. In (a) and (b) the MLR values refer to the normalized MLR regression coefficients. The mean importance indicates the average over all the models, for each parameter, respectively.

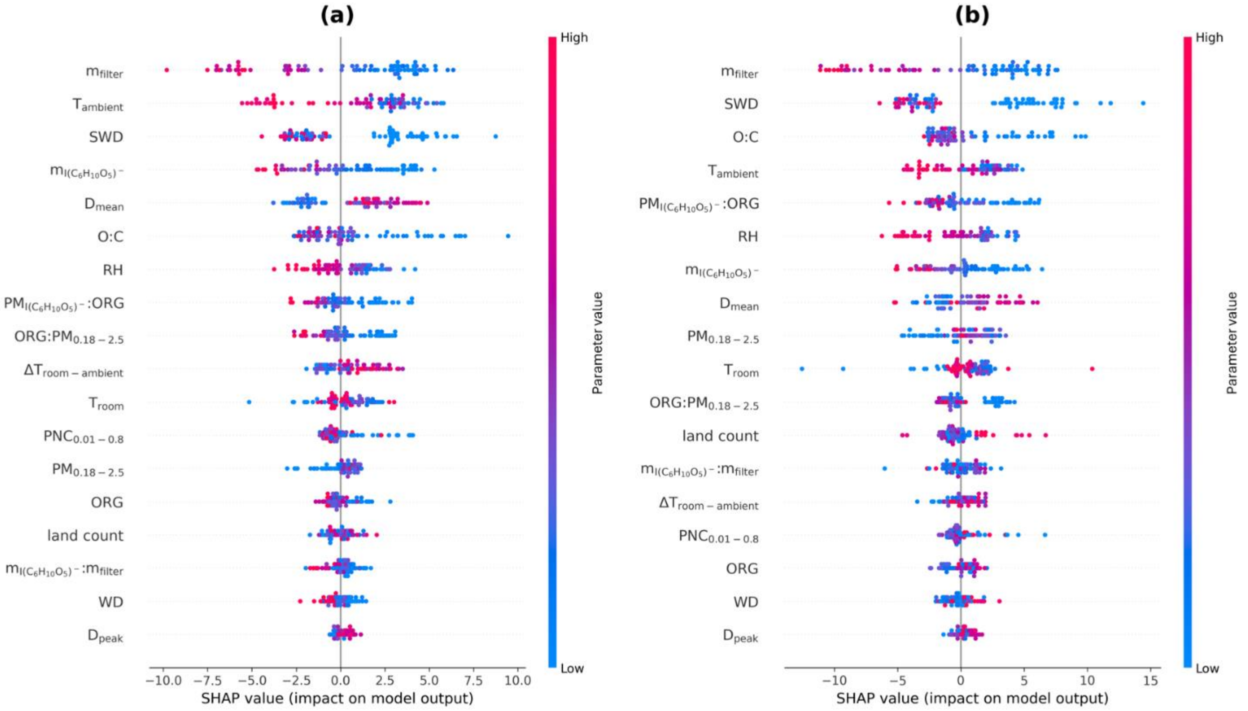

Figure S12. SHAP summary plot, when data from category 6 (Figure 2) is excluded for (a) RF Regressor and (b) XGBoost Regressor after exclusion of Category 6 thermograms. From top to bottom the parameters are sorted according to their overall importance according to SHAP, while the colored dots indicate the direction in which a high (red) or a low (blue) parameter value are related to T_{max}. The dots represent each one prediction of T_{max}. Negative values on the x-axis indicate a decrease in T_{max}, while positive values on the x-axis indicate an increase in T_{max}.

S11 Impact of 18 Parameters on T_{max} Applying Background Correction

The thermograms in the main analysis contain only sample thermograms, where no blank correction is applied, meaning no background signal was subtracted from the sample signals. Considering that some thermograms resemble blank conditions (e.g. those in Category 6), additional analysis was performed to test if a background correction would change the influence of the different parameters on T_{max}. Correction of the sample thermograms was done as follows: For each sample thermogram, a corresponding average blank thermogram was calculated (average of the two closest (in time) blank thermograms) and subsequently subtracted. An example visualizing this correction of the sample thermograms is shown in Figure S13. Thermograms that do not show signal after this background correction were excluded (in total 66). Figure S14 shows the impact of the 18 parameters on T_{max} when using background corrected thermograms, and Figure S15 the corresponding SHAP summary plot. Overall, the relevance of the different parameters on T_{max} is very similar to when using the non-blank corrected thermograms in the main manuscript. The mass on the FIGAERO filter remains the most important factor.

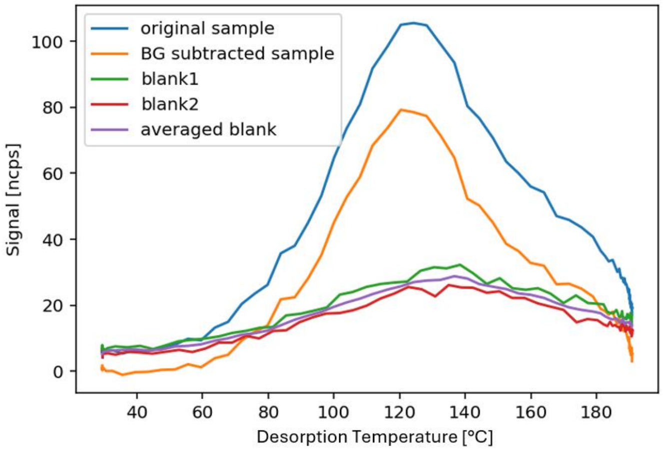

Figure S13. Example of blank-corrected thermograms. The blue thermogram shows the original thermogram sample, while green (blank1) and red (blank2) show the two closest (in time) blank

thermogram signals. The purple line (averaged blanks) shows the averaged blank thermogram using blank1 and blank2. The orange line shows the resulting blank-corrected thermogram (BG subtracted sample).

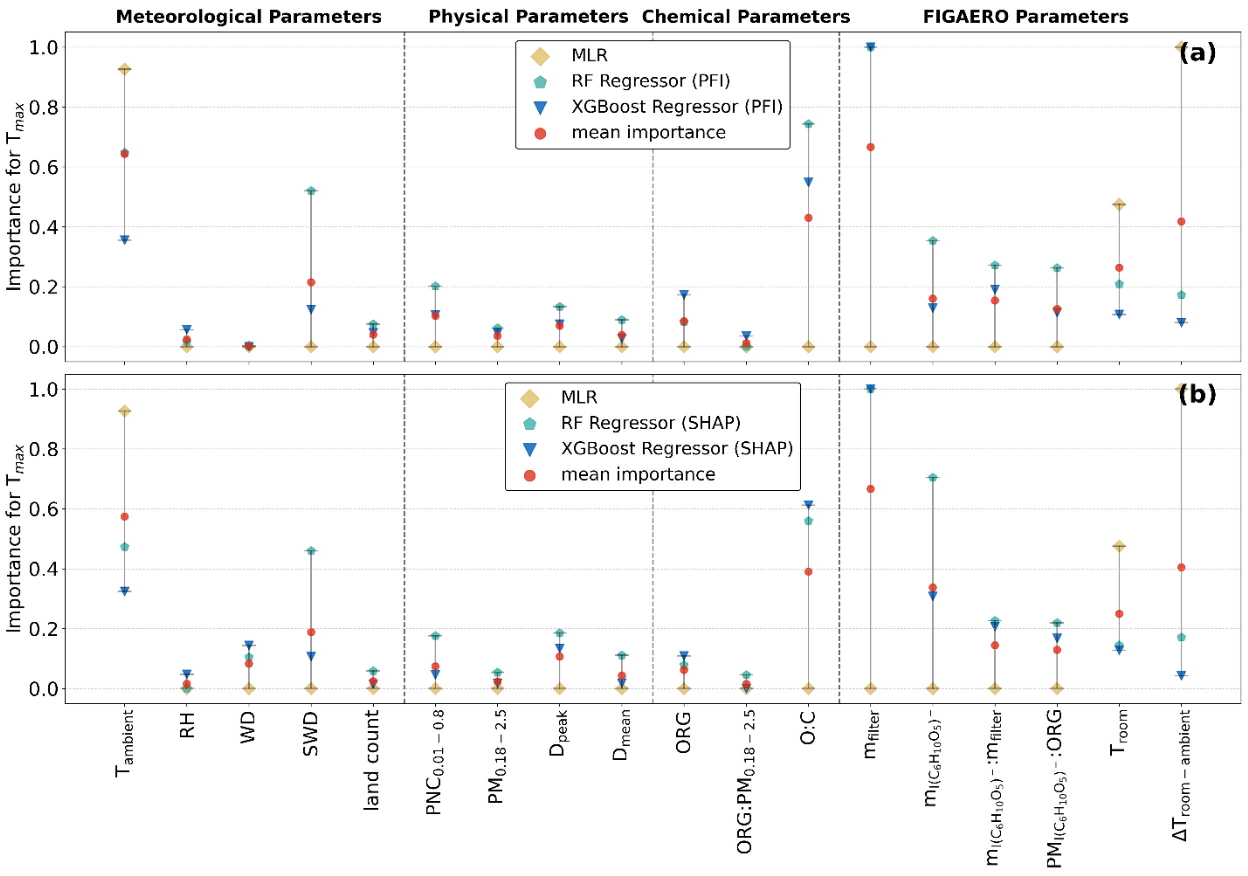

Figure S14. Comparison of the normalized importance of parameters for T_{max} variation, grouped into the categories Meteorological Parameters, Physical Parameters, Chemical Parameters, FIGAERO Parameters, retrieved from the three different models, when using blank-corrected thermograms. To make the models comparable, the importances of the parameters per model were normalized using the MinMaxScaler from the scikit-learn library[6]. (a) shows the normalized importance of the permutation feature importance (PFI), while (b) shows the normalized importance of the SHAP values. In (a) and (b) the MLR values refer to the normalized MLR

regression coefficients. The mean importance indicates the average over all the models, for each parameter, respectively.

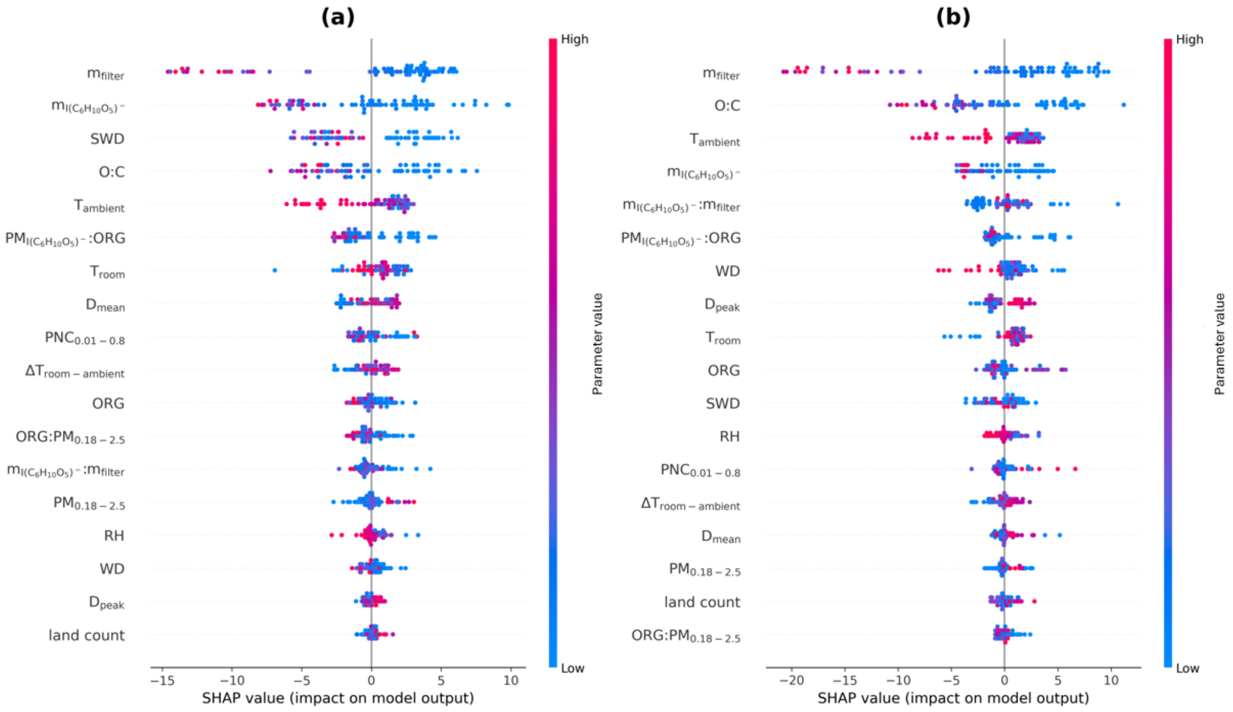

Figure S15. SHAP summary plot, when using blank-corrected thermograms for (a) RF Regressor and (b) XGBoost Regressor for blank-corrected thermograms. From top to bottom the parameters are sorted according to their overall importance according to SHAP, while the colored dots indicate the direction in which a high (red) or a low (blue) parameter value are related to T_{max}. The dots represent each one prediction of T_{max}. Negative values on the x-axis indicate a decrease in T_{max}, while positive values on the x-axis indicate an increase in T_{max}.

S12 Impact of 18 Parameters on T_{max} for other Organic Compounds than Levoglucosan

In the main text, the behavior of levoglucosan thermograms is investigated in great detail. To identify if the observed results are transferable to other organic compounds measured with the FIGAERO-CIMS, two additional compounds are taken for comparison. These two compounds were selected based on their different carbon length and different O/C ratio compared to levoglucosan. The selected compounds were $I(C_3H_4O_4)^-$, most likely malonic acid, and $I(C_9H_{14}O_5)^-$. The thermograms of both compounds were treated in the same way as the thermograms of levoglucosan, i.e., the thermogram samples were used without blank-correction. The thermograms of $I(C_3H_4O_4)^-$ and $I(C_9H_{14}O_5)^-$ are shown in Figure S16 and S17, respectively. Figure S18 presents the impact of the 18 different parameters on T_{max} for $I(C_3H_4O_4)^-$, and Figure S19 the corresponding SHAP summary plot. For $I(C_9H_{14}O_5)^-$ Figure S20 shows the impact of the 18 different parameters on T_{max}, and Figure S21 the corresponding SHAP summary plot. The mass on the FIGAERO filter ranks among the top three most important factors for both compounds. The most impactful parameter for $I(C_3H_4O_4)^-$ thermograms however is the mass of $I(C_3H_4O_4)^-$, and for $I(C_9H_{14}O_5)^-$ thermograms the O/C ratio. Further, both ML models indicate higher T_{max} with lower mass on the filter (Figures S19, S21), which is in line with the result for levoglucosan.

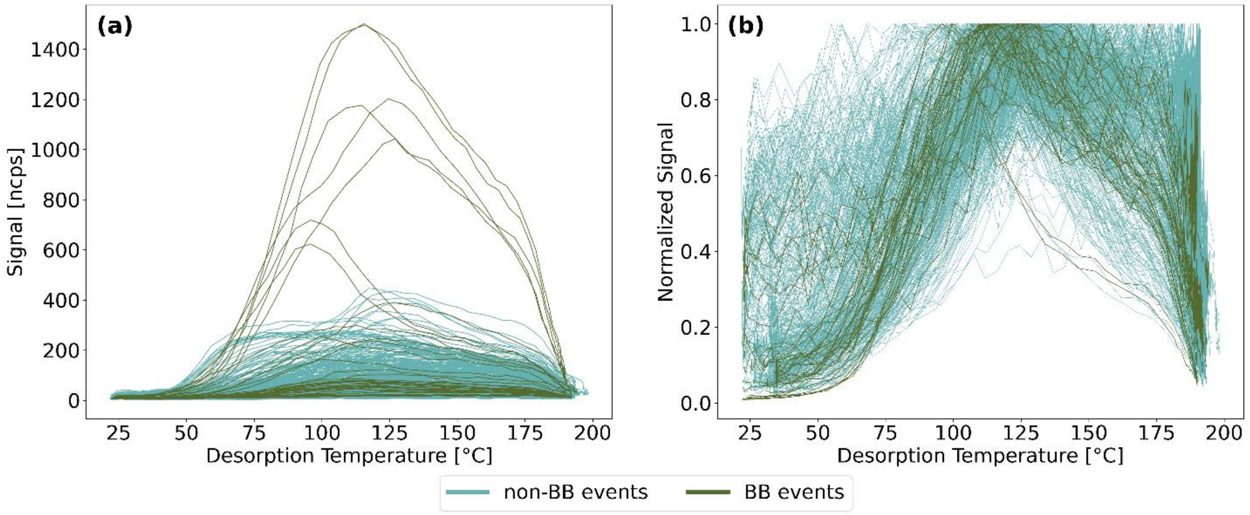

Figure S16. Overview of I($C_3H_4O_4$)$^-$ thermograms as (a) absolute signal and (b) normalized to maximum signal. Those measured during BB events are colored in dark green (BB events) while those from the remaining part of the year (non-BB events) are presented in blue.

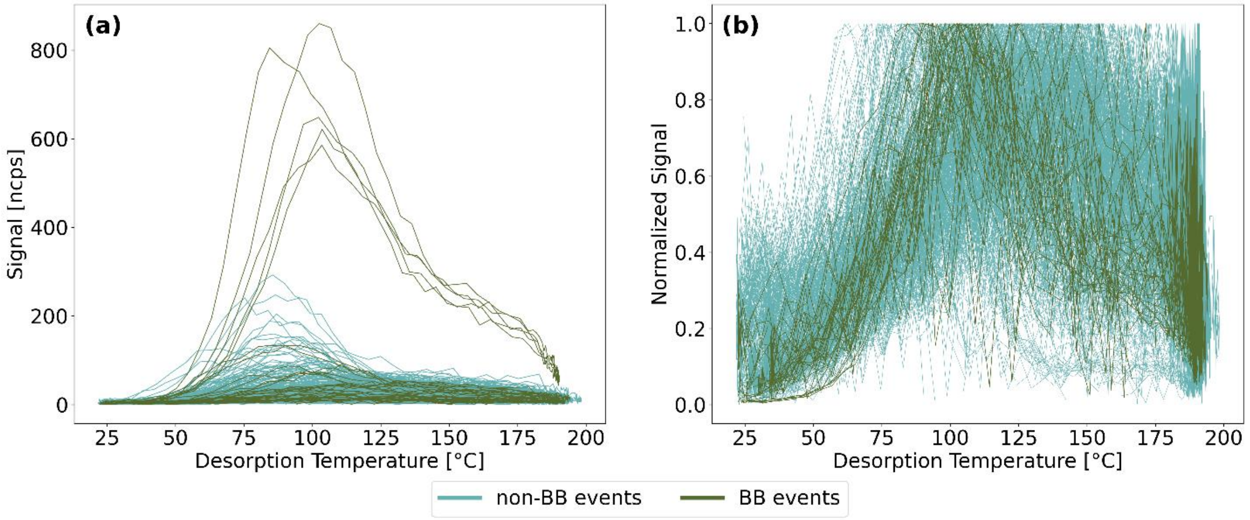

Figure S17. Overview of I($C_9H_{14}O_5$)$^-$ thermograms as (a) absolute signal and (b) normalized to maximum signal. Those measured during BB events are colored in dark green (BB events) while those from the remaining part of the year (non-BB events) are presented in blue.

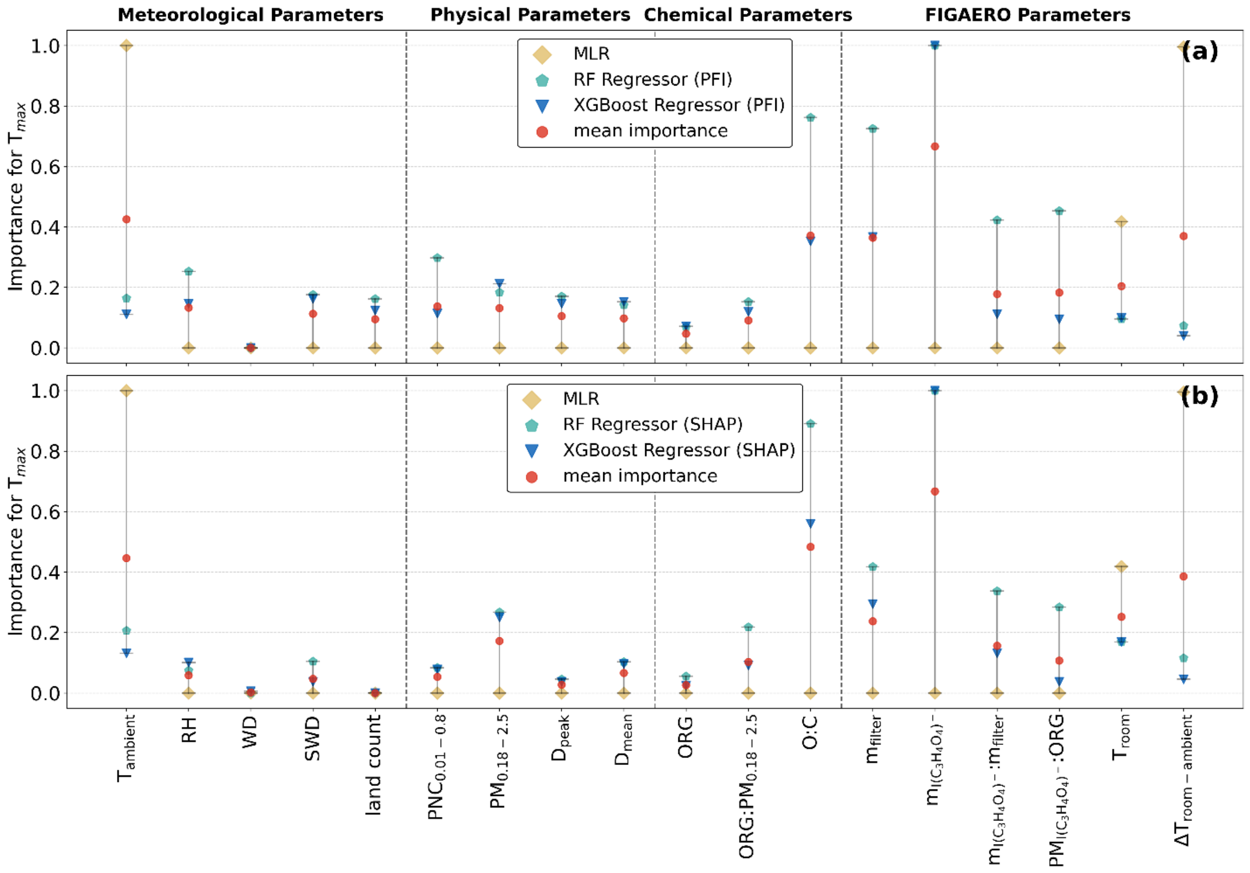

Figure S18. Comparison of the normalized importance of parameters for T_{max} variation, grouped into the categories Meteorological Parameters, Physical Parameters, Chemical Parameters, FIGAERO Parameters, retrieved from the three different models, when using $I(C_3H_4O_4)^-$ thermograms. To make the models comparable, the importances of the parameters per model were normalized using the MinMaxScaler from the scikit-learn library[6]. (a) shows the normalized importance of the permutation feature importance (PFI), while (b) shows the normalized importance of the SHAP values. In (a) and (b) the MLR values refer to the normalized MLR regression coefficients. The mean importance indicates the average over all the models, for each parameter, respectively.

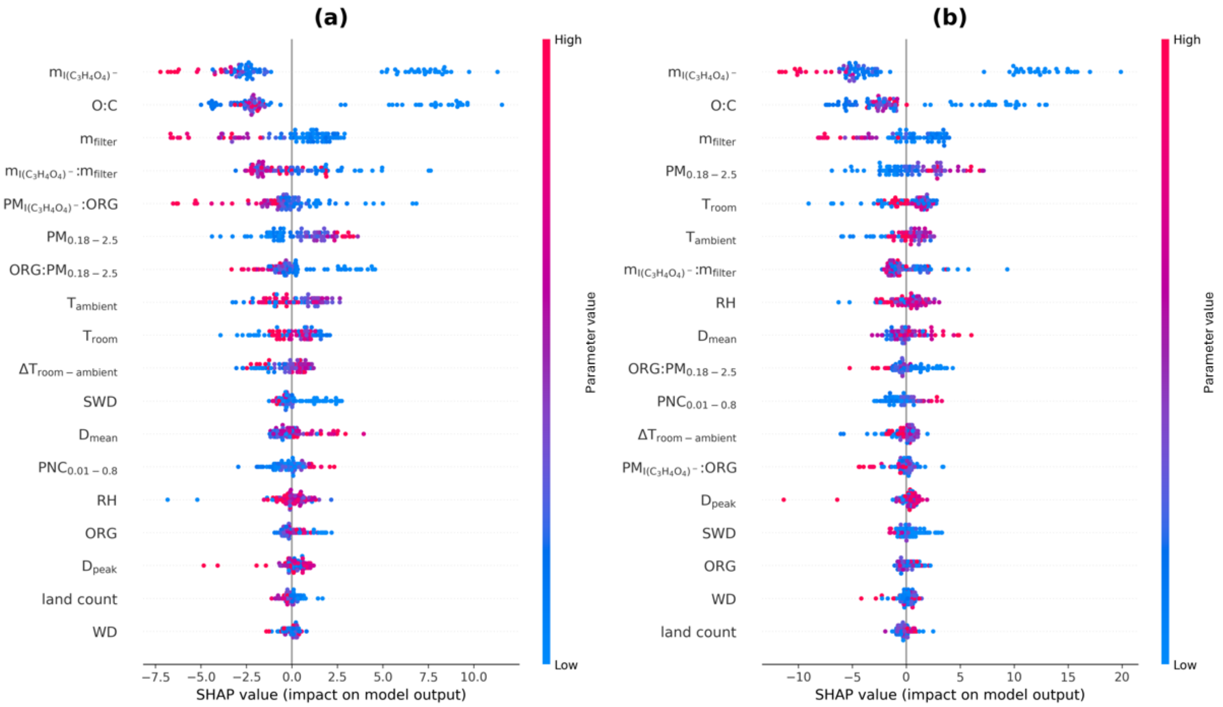

Figure S19. SHAP summary plot for (a) RF Regressor and (b) XGBoost Regressor for T_{max} of $I(C_3H_4O_4)^-$. From top to bottom the parameters are sorted according to their overall importance according to SHAP, while the colored dots indicate the direction in which a high (red) or a low (blue) parameter value are related to T_{max}. The dots represent each one prediction of T_{max}. Negative values on the x-axis indicate a decrease in T_{max}, while positive values on the x-axis indicate an increase in T_{max}.

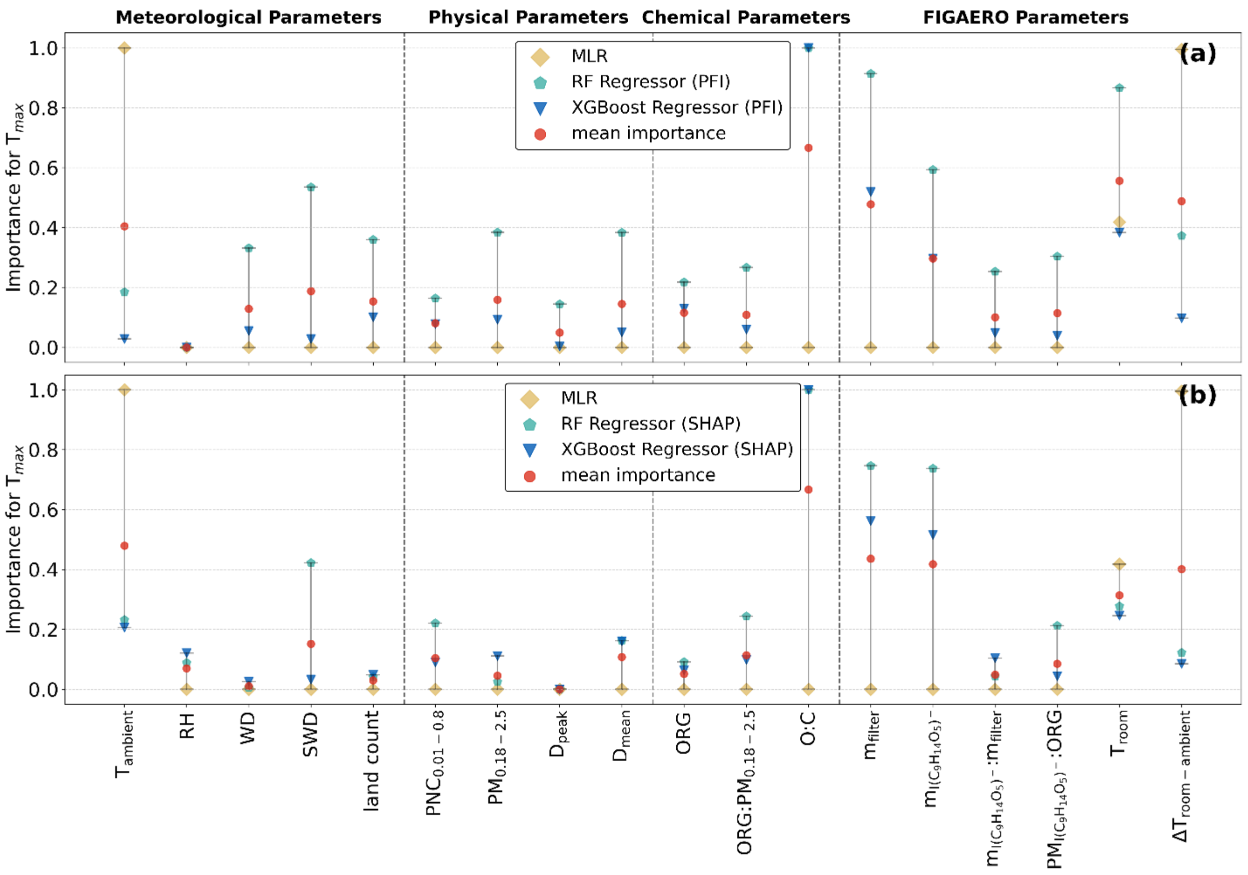

Figure S20. Comparison of the normalized importance of parameters for T_{max} variation, grouped into the categories Meteorological Parameters, Physical Parameters, Chemical Parameters, FIGAERO Parameters, retrieved from the three different models, when using $I(C_9H_{14}O_5)^-$ thermograms. To make the models comparable, the importances of the parameters per model were normalized using the MinMaxScaler from the scikit-learn library[6]. (a) shows the normalized importance of the permutation feature importance (PFI), while (b) shows the normalized importance of the SHAP values. In (a) and (b) the MLR values refer to the normalized MLR regression coefficients. The mean importance indicates the average over all the models, for each parameter, respectively.

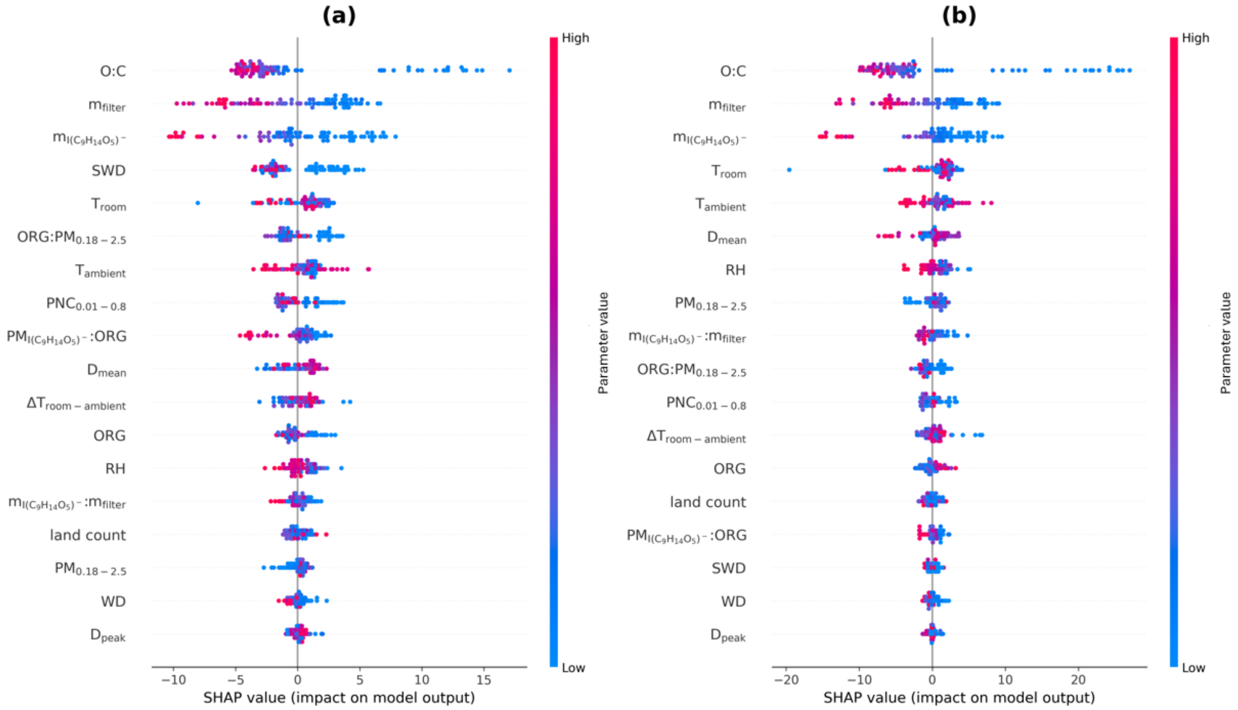

Figure S21. SHAP summary plot for (a) RF Regressor and (b) XGBoost Regressor for T_{max} of $I(C_9H_{14}O_5)^-$. From top to bottom the parameters are sorted according to their overall importance according to SHAP, while the colored dots indicate the direction in which a high (red) or a low (blue) parameter value are related to T_{max}. The dots represent each one prediction of T_{max}. Negative values on the x-axis indicate a decrease in T_{max}, while positive values on the x-axis indicate an increase in T_{max}.

S13 Influence of Inorganic Components on T_{max}

Next to organic species, aerosols in the Arctic can also contain inorganic species such as sea salt (mostly NaCl), which can originate from blowing snow[7] or from sea spray aerosols from oceans and open leads[8]. To investigate the influence of such species on T_{max} of levoglucosan, sodium data was included in the analysis. Due to their extremely low volatility, inorganic salts cannot be measured by FIGAERO-CIMS. Sodium data was downloaded from the EBAS database (https://ebas.nilu.no, last access March 14, 2026)[9] with a daily resolution, derived from offline filter samples and added as an additional parameter in the regression models. Figure S22 shows the impact of sodium and the other 18 parameters on T_{max} of levoglucosan thermograms and Figure S23 the corresponding SHAP summary plots. They show that sodium is negligible for influencing T_{max}, and that parameters related to FIGAERO-CIMS filter mass remain the most important parameters, emphasizing the robustness of our results.

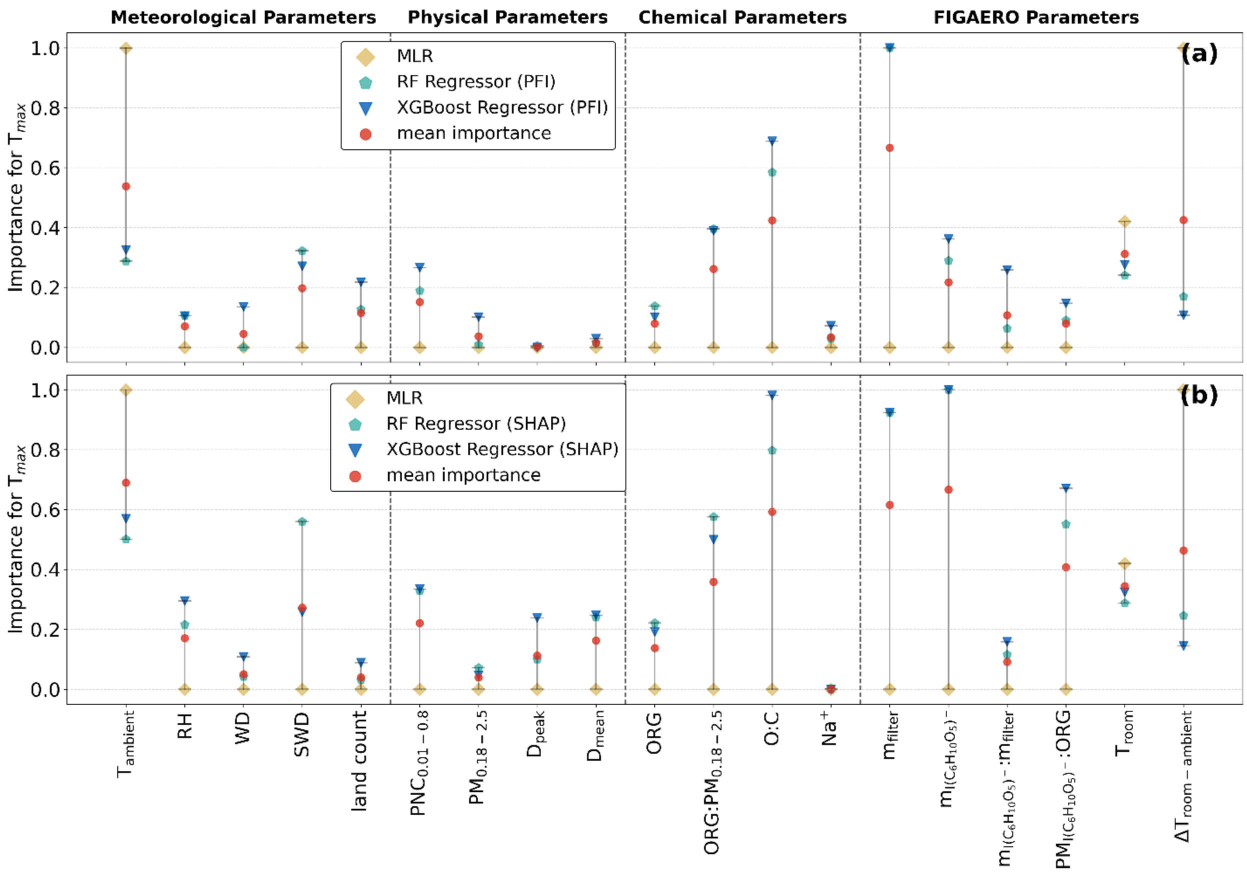

Figure S22. Comparison of the normalized importance of parameters for T_{max} variation, grouped into the categories Meteorological Parameters, Physical Parameters, Chemical Parameters, FIGAERO Parameters, retrieved from the three different models, when an inorganic species is included as additional parameter (sodium: Na^+). To make the models comparable, the importances of the parameters per model were normalized using the MinMaxScaler from the scikit-learn library[6]. (a) shows the normalized importance of the permutation feature importance (PFI), while (b) shows the normalized importance of the SHAP values. In (a) and (b) the MLR values refer to the normalized MLR regression coefficients. The mean importance indicates the average over all the models, for each parameter, respectively.

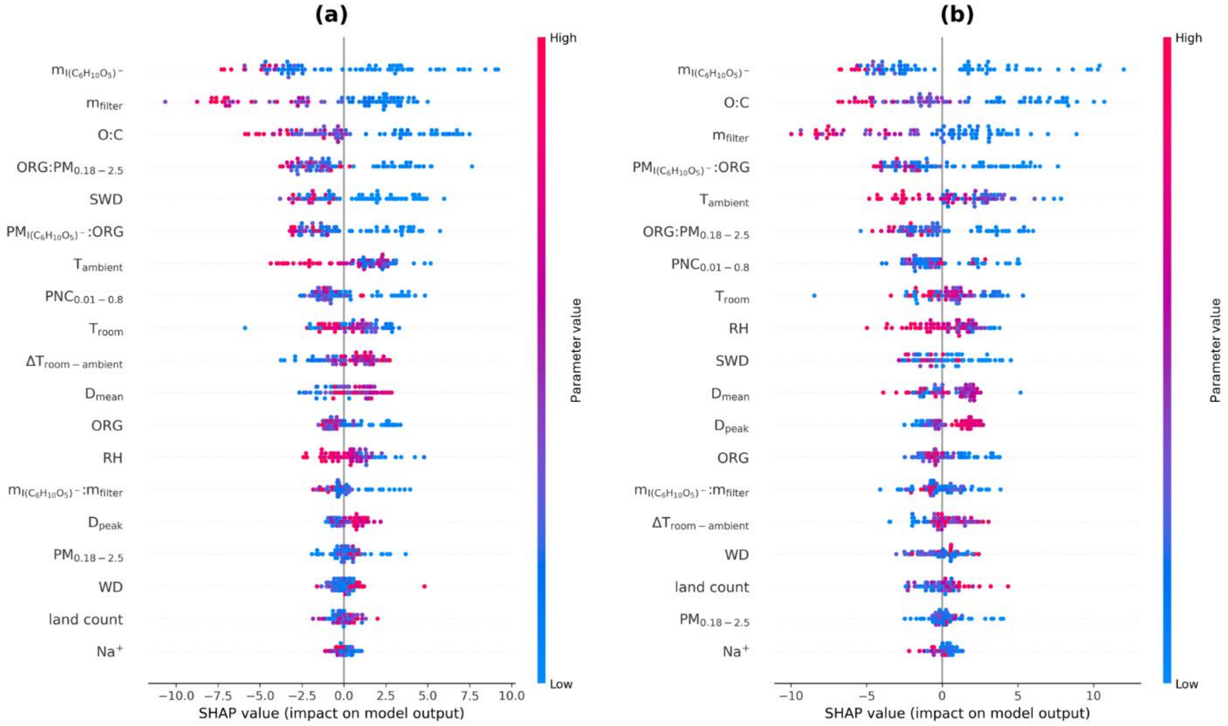

Figure S23. SHAP summary plot for (a) RF Regressor and (b) XGBoost Regressor including Na^+ as a parameter. From top to bottom the parameters are sorted according to their overall importance according to SHAP, while the colored dots indicate the direction in which a high (red) or a low (blue) parameter value are related to T_{max}. The dots represent each one prediction of T_{max}. Negative values on the x-axis indicate a decrease in T_{max}, while positive values on the x-axis indicate an increase in T_{max}.

S14 Relation of T_{max} and FIGAERO Mass Loading

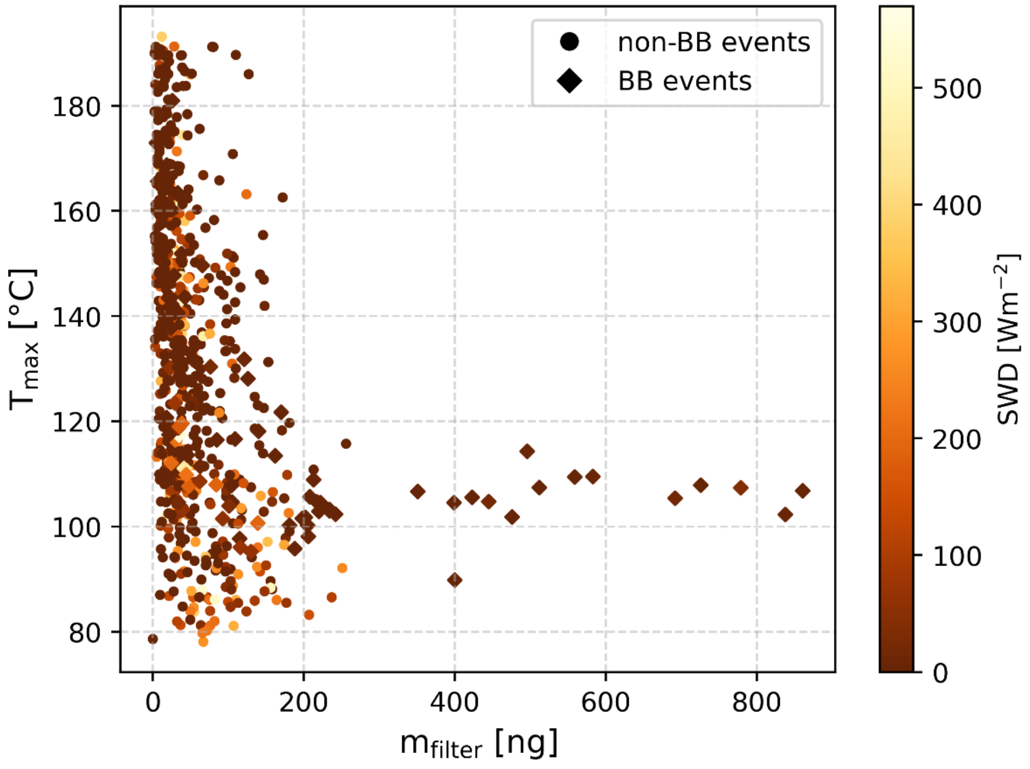

Figure S24. T_{max} as function of m_{filter}, color coded by SWD. The data corresponding to non-BB events are presented in circles, whereas the BB events are shown as diamonds. The data presented here include all sample thermograms (n = 745) from 2020, while the main manuscript (Figure 5) shows only the data that were used for the models. Since the number of datapoints in the main manuscript was determined based on the availability of all the other parameter data, there are fewer datapoints in the main manuscript figure.

AUTHOR INFORMATION

Corresponding Author

Claudia Mohr, Claudia.mohr@psi.ch.

Present Addresses

§ Karolina Siegel: Swedish Meteorological and Hydrological Institute (SMHI), Norrköping, 60176, Sweden

Author Contributions

‡These authors contributed equally.

REFERENCES

(1) Gramlich, Y.; Siegel, K.; Haslett, S. L.; Cremer, R. S.; Lunder, C.; Kommula, S. M.; Buchholz, A.; Yttri, K. E.; Chen, G.; Krejci, R.; Zieger, P.; Virtanen, A.; Riipinen, I.; Mohr, C. Impact of Biomass Burning on Arctic Aerosol Composition. *ACS Earth Space Chem.* **2024**, *8* (5), 920–936. https://doi.org/10.1021/acsearthspacechem.3c00187.

(2) Lopez-Hilfiker, F. D.; Iyer, S.; Mohr, C.; Lee, B. H.; D'Ambro, E. L.; Kurtén, T.; Thornton, J. A. Constraining the Sensitivity of Iodide Adduct Chemical Ionization Mass Spectrometry to Multifunctional Organic Molecules Using the Collision Limit and Thermodynamic Stability of Iodide Ion Adducts. *Atmospheric Meas. Tech.* **2016**, *9* (4), 1505–1512. https://doi.org/10.5194/amt-9-1505-2016.

(3) Freitas, G. P.; Adachi, K.; Conen, F.; Heslin-Rees, D.; Krejci, R.; Tobo, Y.; Yttri, K. E.; Zieger, P. Regionally Sourced Bioaerosols Drive High-Temperature Ice Nucleating Particles in the Arctic. *Nat. Commun.* **2023**, *14* (1), 5997. https://doi.org/10.1038/s41467-023-41696-7.

(4) Savitzky, Abraham.; Golay, M. J. E. Smoothing and Differentiation of Data by Simplified Least Squares Procedures. *Anal. Chem.* **1964**, *36* (8), 1627–1639. https://doi.org/10.1021/ac60214a047.

(5) Gramlich, Y.; Siegel, K.; Haslett, S. L.; Freitas, G.; Krejci, R.; Zieger, P.; Mohr, C. Revealing the Chemical Characteristics of Arctic Low-Level Cloud Residuals – in Situ Observations from a Mountain Site. *Atmospheric Chem. Phys.* **2023**, *23* (12), 6813–6834. https://doi.org/10.5194/acp-23-6813-2023.

(6) Pedregosa, F.; Varoquaux, G.; Gramfort, A.; Michel, V.; Thirion, B.; Grisel, O.; Blondel, M.; Prettenhofer, P.; Weiss, R.; Dubourg, V.; Vanderplas, J.; Passos, A.; Cournapeau, D.; Brucher, M.; Perrot, M.; Duchesnay, E. Scikit-Learn: Machine Learning in Python. *JMLR* **2011**, *12*, 2825–2830.

(7) Gong, X.; Zhang, J.; Croft, B.; Yang, X.; Frey, M. M.; Bergner, N.; Chang, R. Y.-W.; Creamean, J. M.; Kuang, C.; Martin, R. V.; Ranjithkumar, A.; Sedlacek, A. J.; Uin, J.; Willmes, S.; Zawadowicz, M. A.; Pierce, J. R.; Shupe, M. D.; Schmale, J.; Wang, J. Arctic Warming by Abundant Fine Sea Salt Aerosols from Blowing Snow. *Nat. Geosci.* **2023**, *16* (9), 768–774. https://doi.org/10.1038/s41561-023-01254-8.

(8) Kirpes, R. M.; Bonanno, D.; May, N. W.; Fraund, M.; Barget, A. J.; Moffet, R. C.; Ault, A. P.; Pratt, K. A. Wintertime Arctic Sea Spray Aerosol Composition Controlled by Sea Ice Lead Microbiology. *ACS Cent. Sci.* **2019**, *5* (11), 1760–1767. https://doi.org/10.1021/acscentsci.9b00541.

(9) Tørseth, K.; Aas, W.; Breivik, K.; Fjæraa, A. M.; Fiebig, M.; Hjellbrekke, A. G.; Lund Myhre, C.; Solberg, S.; Yttri, K. E. Introduction to the European Monitoring and Evaluation Programme (EMEP) and Observed Atmospheric Composition Change during 1972–2009. *Atmospheric Chem. Phys.* **2012**, *12* (12), 5447–5481. https://doi.org/10.5194/acp-12-5447-2012.
